# A Comprehensive Analysis of Epoxide Hydrolase 2 (EPHX2) in Pan‐Cancer

**DOI:** 10.1002/cnr2.70188

**Published:** 2025-03-24

**Authors:** Weiquan Hu, Xiaoli Ding, Xiangsheng Wu, Xuxiang Xi, Jing Xu, Shengyun Dai, Jing Chen, Suping Hu, Qinfei Zhao, Fangfang Chen

**Affiliations:** ^1^ Department of Joint Surgery Ganzhou People's Hospital Ganzhou Jiangxi China; ^2^ Department of Laboratory Medicine First Affiliated Hospital of Gannan Medical University Ganzhou Jiangxi China; ^3^ Department of Orthopaedic Surgery Sun Yat‐Sen Memorial Hospital, Sun Yat‐Sen University Guangzhou Guangdong China; ^4^ National Institutes for Food and Drug Control Beijing China; ^5^ Department of Emergency First Affiliated Hospital of Gannan Medical University Ganzhou Jiangxi China; ^6^ The First School of Clinical Medicine, Southern Medical University Guangzhou Guangdong China; ^7^ Jinling Hospital, Affiliated Hospital of Medical School, Nanjing University Nanjing Jiangsu China

**Keywords:** EPHX2, immune checkpoint blockade (ICB), immune infiltration, immunotherapy, pan‐cancer, prognosis

## Abstract

**Background and Aims:**

Epoxide hydrolase 2 (EPHX2) regulates lipid signaling across various metabolites by encoding soluble epoxide hydrolase. However, its mechanisms and implications in human malignancies remain unknown. This research aimed to detail the prognostic landscape of EPHX2 in pan‐cancer and explore its potential relationship with immune infiltration in the tumor microenvironment.

**Methods:**

Herein, multiple bioinformatics tools were used to comprehensively evaluate the expression, diagnostic, and prognostic significance of EPHX2 and its roles in the tumor immune microenvironment in human cancers. The underlying EPHX2‐associated signaling pathways in cancers were investigated by gene set variation analysis (GSVA). TIDE, GDSC, and CTRP databases were applied to predict the response of EPHX2 to immunotherapy and sensitivity to small molecule drugs. Furthermore, EPHX2 expression was also validated by qPCR experiments in various cancer cell lines.

**Results:**

Overall results revealed significant down‐regulation of EPHX2 mRNA expression in most tumors. Despite its high predictive significance across cancers, EPHX2 played a protective or detrimental effect in distinct types of cancers. EPHX2 proved to be a valuable diagnostic biomarker in a range of tumor types, particularly in kidney renal clear cell carcinoma, cervical squamous cell carcinoma, and endocervical adenocarcinoma. Genetic alterations of EPHX2 in 33 tumors were also investigated. EPHX2 expression was significantly linked to immune cell infiltrations (particularly tumor‐associated macrophages), tumor mutation burden, microsatellite instability, immune modulators, and immunotherapeutic biomarkers. Single‐cell sequencing and GSVA highlighted the relevance of EPHX2 in regulating various cancer‐related biological processes, including cell cycle and apoptosis. In this view, targeting EPHX2‐dependent signaling could be a promising therapeutic strategy for tumor immunotherapy.

**Conclusion:**

EPHX2 may serve as a potential molecular biomarker for diagnosis and prognosis in pan‐cancer and could become a novel therapeutic target for various cancers.

**Abbreviations**
ACC: adrenocortical carcinoma
AUC: area under the ROC curves
BLCA: bladder urothelial carcinoma
BRCA: breast cancer
CAN: copy number alteration
CESC: cervical squamous cell carcinoma and endocervical adenocarcinoma
CHOL: cholangiocarcinoma
COAD: colon adenocarcinoma
CRC: colorectal cancer
ccRCC: clear cell renal cell carcinoma
CPTAC: clinical proteomic tumor analysis consortium
CTRP: Cancer Therapeutics Response Portal
DSS: disease‐specific survival
DFS: disease‐free survival
DLBC: lymphoid neoplasm diffuse large B‐cell lymphoma
ESCA: esophageal carcinoma
EPHX2: epoxide hydrolase 2
FS: progression‐free survival
GBM: glioblastoma multiforme
GEPIA2: gene expression profiling interactive analysis 2
GDSC: Genomics of Drug Sensitivity in Cancer
GSVA: gene set variation analysis
HNSC: head and neck squamous cell carcinoma
HPA: Human Protein Atlas
HR: hazard ratio
IC50: half‐maximal inhibitory concentration
ICBs: immune checkpoint blockade
ICPs: immune checkpoint genes
KICH: kidney chromophobe
KIRC: kidney renal clear cell carcinoma
KIRP: kidney renal papillary cell carcinoma
KM: Kaplan–Meier
LAML: acute myeloid leukemia
LUAD: lung adenocarcinoma
LUSC: lung squamous cell carcinoma
LIHC: liver hepatocellular carcinoma
LGG: brain lower grade glioma
MB: megabase
MESO: mesothelioma
MMR: mismatch repair
MSI: microsatellite instability
OS: overall survival
OV: ovarian serous cystadenocarcinoma
PAAD: pancreatic adenocarcinoma
PCPG: paraganglioma
PFS: progression‐free survival
PRAD: prostate adenocarcinoma
PPI: protein–protein interaction
qRT‐PCR: quantitative real‐time polymerase chain reaction
RB: retinoblastoma
READ: rectum adenocarcinoma
SARC: sarcoma
SD: standard deviations
SKCM: skin cutaneous melanoma
STAD: stomach adenocarcinoma
TGCT: testicular germ cell tumors
TIDE: track INDELs with the decomposition
TME: tumor microenvironment
TMB: tumor mutation burden
THCA: thyroid carcinoma
THYM: thymoma
t‐SNE: T‐distributed stochastic neighbor embedding
UCEC: uterine corpus endometrial carcinoma
UCS: uterine carcinosarcoma
UVM: uveal melanoma

## 1 Introduction

Cancer has become one of the leading causes of death worldwide and significantly affects quality of life [1, 2]. Despite advances in cancer research, effective treatments remain limited [3, 4], making the identification of key pan‐cancer genes crucial for understanding cancer initiation, progression, and metastasis [5, 6]. Tumor immunity significantly influences tumor recurrence and poor prognosis, necessitating the study of its mechanisms.

Epoxide hydrolase 2 (EPHX2) encodes soluble epoxide hydrolase, an enzyme involved in the degradation of endogenous lipid epoxide [7, 8, 9], particularly inactivating epoxyeicosatrienoic acids [10]. EPHX2 has been extensively studied for its role in cardiovascular diseases [11], metabolic disorders [12], and inflammatory conditions [13]. Recently, research has begun to uncover its significance in cancer [14, 15]. The enzyme's ability to modulate lipid signaling pathways suggests it might influence tumor growth and progression [16]. Abnormal EPHX2 expression has been observed in various cancers. For instance, decreased EPHX2 expression has been linked to poor prognosis in liver hepatocellular carcinoma (LIHC) [15], and has been identified as a potential tumor suppressor in cervical squamous cell carcinoma [17]. Despite these insights, most studies have focused on EPHX2's role in specific cancer types. Comprehensive pan‐cancer analyses evaluating EPHX2's prognostic significance and biological functions across various tumors are limited. Such studies are essential for fully understanding the diverse roles of EPHX2 in cancer and exploring its potential as a therapeutic target.

Our study sought to fill this gap by systematically evaluating the predictive value of EPHX2 in pan‐cancer using multiple bioinformatic approaches. Leveraging publicly available databases, we investigated the expression profiles at both tumor tissue and single‐cell sequencing levels. To ensure the robustness of our findings, we performed quantitative real‐time polymerase chain reaction (qRT‐PCR) experiments to confirm EPHX2 gene expression levels in various cancer cell lines. Additionally, gene set variation analysis (GSVA) was performed to elucidate the biological role of EPHX2 across 33 tumor types. Our research systematically explored the correlations of EPHX2 expression with clinical prognosis, clinical characteristics, genetic alterations, immune infiltrations within the tumor microenvironment (TME), tumor mutation burden (TMB), microsatellite instability (MSI), immune modulators, cancer immunotherapy response, and drug sensitivity in 33 cancers, providing valuable insights into potential therapeutic strategies.

## 2 Materials and Methods

### 2.1 Gene Expression Analysis

EPHX2 gene expression in different cancer types was investigated using the TIMER (http://timer.cistrome.org/) database. The RNAseq data from TCGA, processed uniformly by the toiling method, were analyzed and visualized using R version 4.0.2 software and the ggplot2 package [18]. The UALCAN portal (http://ualcan.path.uab.edu/analysis‐prot.html), based on the clinical proteomic tumor analysis consortium (CPTAC) database, was used to identify total protein expression levels of EPHX2 in six available tumor datasets, including breast cancer (BRCA), ovarian cancer, colon cancer, clear cell renal cell carcinoma (ccRCC), uterine corpus endometrial carcinoma (UCEC), and lung adenocarcinoma (LUAD). The Human Protein Atlas (HPA) (http://www.proteinatlas.org/) was further employed to assess changes in EPHX2 protein expression. Moreover, the gene expression profiling interactive analysis 2 (GEPIA2) web server (http://gepia2.cancer‐pku.cn/#analysis) was used to create violin plots of EPHX2 expression across various pathological stages of all tumors in the TCGA database.

### 2.2 Survival Prognosis and the Receiver Operating Characteristic Curve Analysis

Kaplan–Meier (KM) analyses were performed to determine overall survival (OS) and disease‐specific survival (DSS) of patients in the TCGA cohort. Univariate Cox regression analyses were performed to assess the prognostic significance of EPHX2 on OS, DSS, disease‐free survival (DFS), and progression‐free survival (PFS) in pan‐cancer. Data from the PrognoScan website (http://www.prognoscan.org/) were also retrieved from TCGA and GEO databases. A *p*‐value of 0.05 denoted a significantly positive prognostic value. The area under the receiver operating characteristic (ROC) curves (AUCs) was calculated to assess the diagnostic and prognostic significance of EPHX2 in cancers.

### 2.3 Genetic Alteration Analysis

EPHX2 genetic alterations in TCGA cancers were assessed using cBioPortal (https://www.cbioportal.org/). Furthermore, R language and packages were employed to analyze and visualize the correlations of EPHX2 with copy number change (CAN) and DNA Methylation data from TCGA.

### 2.4 Association Between EPHX2 Expression and the TME in Pan‐Cancer

The immunological score of each sample was inferred using the ESTIMATE algorithm [19]. Following that, the single‐sample gene set enrichment analysis (ssGSEA) [20], and CIBERSORT [21] algorithms were used to determine the relative fractions of immune infiltrations. TMB was defined as the total number of somatic, coding, base substitutions, and indel mutations per megabase (Mb) of the examined genome. To determine the TMB per Mb, the total number of mutations identified was divided by the 38 Mb exome size. The MSI score of each sample in the TCGA database was determined using previously published data. The potential links between EPHX2 expression and immunomodulators (immunosuppressants, immunostimulants, and MHC molecules) were explored using the TISIDB website (http://cis.hku.hk/TISIDB/index.php). The expression levels of mismatch repair (MMR) and immune checkpoint genes (ICPs) were examined using TCGA data.

### 2.5 The Prediction of Immune Checkpoint Blockade Response

The potential immune checkpoint blockade (ICB) response was predicted using the Track INDELs with the DEcomposition (TIDE) algorithm. TIDE integrated a set of gene expression markers to evaluate two mechanisms of tumor immune evasion: tumor‐infiltrating cytotoxic T‐lymphocyte dysfunction and immunosuppressive factor rejection of cytotoxic T‐lymphocytes. Higher scores indicated poorer efficacy of ICB therapy and shorter survival following treatment.

### 2.6 Single‐Cell Sequencing

CancerSEA (http://biocc.hrbmu.edu.cn/CancerSEA/) [22], a specialized single‐cell sequencing database, was used to investigate the functional status of cancer cells at the single‐cell level. The link between EPHX2 expression and distinct tumor functions was analyzed using single‐cell sequencing data. T‐Distributed stochastic neighbor embedding (t‐SNE) diagrams revealed the EPHX2 expression profiles of single cells from TCGA samples.

### 2.7 Protein–Protein Interaction Network Construction

In this study, the EPHX2 protein–protein interaction (PPI) was analyzed using the GeneMANIA (http://genemania.org/) and STRING (https://string‐db.org/) databases.

### 2.8 Gene‐Set Variation Analysis

The GSVA was performed to identify potential EPHX2 signaling pathways using a HALLMARK gene set file from the MSigDB database [20]. A heat map was created based on the results of the R packages “clusterProfiler” and “GSVA.”

### 2.9 Drug Sensitivity Analysis

Gene expression profiles and drug sensitivity data were retrieved from the Genomics of Drug Sensitivity in Cancer (GDSC) and Cancer Therapeutics Response Portal (CTRP) databases. The association between gene expression and half‐maximal inhibitory concentration (IC50) values of drugs was evaluated using Pearson's correlation analysis.

### 2.10 Cell Culture

Human ureter epithelial cell line SV‐HUC‐1, human bladder cancer cell lines T24 and 5637, human renal proximal convoluted tubular epithelial cell line HK‐2, human renal cancer cell lines A‐498 and 786‐O, human intestinal epithelial cell line HIEC, human colon carcinoma cells HT29, HCT8, and HCT116, human hepatocyte cell line L‐O2, human hepatoma cell line HUH‐7, HepG2, and MHCC‐97H were obtained from our research group and preserved at the Department of Laboratory Medicine, First Affiliated Hospital of Gannan Medical University. SV‐HUC‐1, A‐498, HIEC, HT29, HUH‐7, HepG2, and MHCC‐97H cells were incubated in DMEM, while T24, 5637, HK‐2, 786‐O, HCT8, HCT116, and L‐O2 cells were incubated in RPMI‐1640. All media were supplemented with 10% FBS (TransGen) and 1% antibiotics (100 U/mL penicillin and 100 μg/mL streptomycin) (Solarbio, China). All cells were maintained at 37°C in a 5% CO_2_ incubator (MCO‐170AICUVHL‐PC, Phcbi, Japan).

### 2.11 RNA Isolation and qRT‐PCR

RNA was isolated from the cells utilizing the TransZol Up reagent kit (TRANS, Beijing, China). The concentration and quality of the extracted RNA were assessed using a spectrophotometer (Nanodrop One, ThermoFisher Scientific), ensuring an absorbance ratio (260/280) greater than 1.8. The RNA was then reverse‐transcribed into cDNA employing the PrimeScript RT reagent Kit (Perfect Real Time) (Takara). The qRT‐PCR experiment was subsequently performed using the PerfectStart TM Green qPCR SuperMix kit (TransGens, China) on an Applied Biosystems QuantStudio 5 thermal cycler (ThermoFisher Scientific). The relative abundance of mRNA was determined employing the comparative cycle threshold method (2^−∆∆CT^), with hACTIN serving as an internal control. All qPCR experiments were conducted in triplicate, and the results are presented as mean values ± standard deviations (SD) derived from three independent experiments. The primers used in this study were designed using Primer website tools and synthesized by Generay Biotechnology Co. (Shanghai, China). Primer sequences are as follows: EPHX2 (forward: 5′‐GTGCTGAGAGAGATGGCCTG‐3′, reverse: 5′‐CATTCCCACCTGACACGACT‐3′) and hACTIN (forward: 5′‐CATGTACGTTGCTATCCAGGC‐3′, reverse: 5′‐CTCCTTAATGTCACGCACGAT‐3′).

### 2.12 Statistical Analysis

Cancer patients were classified into high and low EPHX2 expression groups based on the median EPHX2 expression value. KM survival curves were constructed to assess OS and DS. Differences between the groups were analyzed using the log‐rank test, and hazard ratios (HRs) were calculated using Cox proportional hazards regression models. The comparison of baseline data between groups was performed using the Chi‐square test when all expected frequencies exceeded 5 and the total sample size was 40 or greater. When expected frequencies ranged from 1 to less than 5, with a total sample size of 40 or greater, the Chi‐square test with Yates' correction was applied. Pearson's correlation coefficients were used to assess the relationships between EPHX2 expression and continuous variables, such as TMB, MSI, immune cell infiltration, and immunomodulators. Differences in continuous variables were compared using the Wilcoxon signed‐rank test. Multiple hypothesis testing was adjusted using the Benjamini–Hochberg method. For the RT‐qPCR data analysis, a one‐way analysis of variance (ANOVA) was employed to assess the overall differences among independent groups. All statistical analyses were performed in R version 4.0.2. *p* values ≤ 0.05 denoted statistical significance.

## 3 Results

### 3.1 The Different Expression Profiles of EPHX2 in Human Pan‐Cancer

**FIGURE 1:**
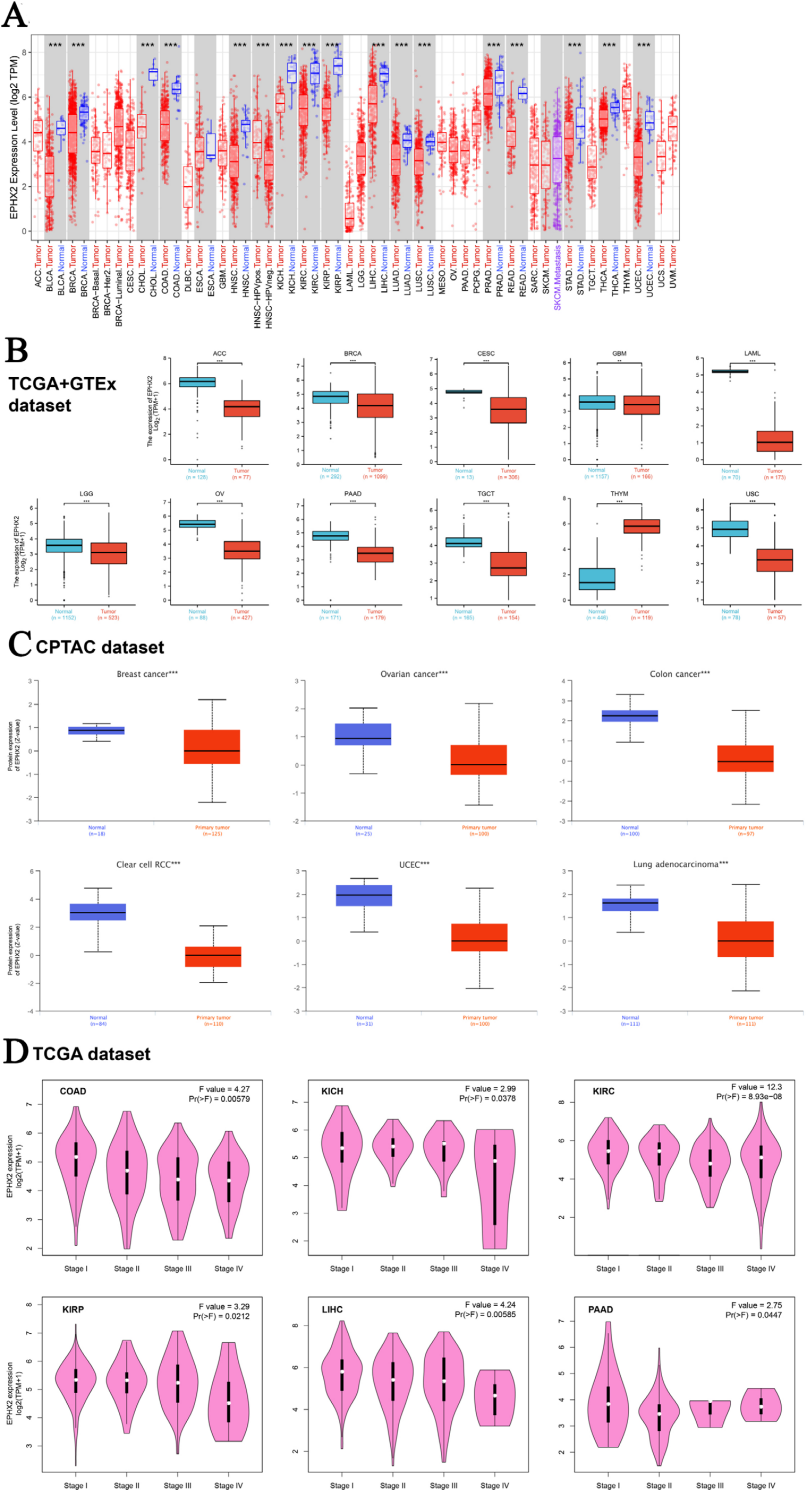
Expression levels of EPHX2 in human cancers. (A) The EPHX2 expression status in diverse cancers or specific cancer subtypes using the TIMER2 database (***p < 0.001). (B) The expression differences of EPHX2 in ACC, BRCA, CESC, GBM, LAML, LGG, OV, PAAD, TGCT, THYM, and USC from the TCGA and GTEx databases (**p < 0.01; ***p < 0.001). (C) The total‐protein expression levels of EPHX2 in breast cancer, ovarian cancer, colon cancer, clear cell RCC, UCEC, and LUAD were analyzed using the CPTAC data portal (***p < 0.001). (D) The correlations between the EPHX2 expression levels and the pathological stages were analyzed using the GEPIA2 tool.

In our study, the TIMER2.0, UALCAN, HPA, and GEPIA2.0 databases were used to analyze EPHX2 expression in tumors and corresponding normal tissues, providing a mutually corroborative comparison of the data. Initially, we assessed EPHX2 mRNA expression in pan‐cancer using the TIMER2 method. As illustrated in Figure 1A, EPHX2 expression was significantly downregulated in numerous cancer types, including bladder urothelial carcinoma (BLCA), BRCA, cholangiocarcinoma (CHOL), head and neck squamous cell carcinoma (HNSC), colon adenocarcinoma (COAD), kidney renal papillary cell carcinoma (KIRP), kidney chromophobe (KICH), kidney renal clear cell carcinoma (KIRC), LIHC, LUAD, lung squamous cell carcinoma (LUSC), prostate adenocarcinoma (PRAD), rectum adenocarcinoma (READ), stomach adenocarcinoma (STAD), thyroid carcinoma (THCA), and UCEC. The TCGA and GTEx databases were also used to assess the differences in EPHX2 expression (Figure 1B). Further validation results indicated that EPHX2 expression levels were profoundly decreased in adrenocortical carcinoma (ACC), BRCA, cervical squamous cell carcinoma and endocervical adenocarcinoma (CESC), glioblastoma multiforme (GBM), brain lower grade glioma (LGG), ovarian serous cystadenocarcinoma (OV), pancreatic adenocarcinoma (PAAD), testicular germ cell tumors (TGCT), and uterine carcinosarcoma (UCS), while notably upregulated in thymoma (THYM) (Figure 1B). Figure S1 illustrates the differential expression of EPHX2 in tumor and paired non‐tumor tissues across various cancers in the TCGA database. Overall, EPHX2 was significantly more weakly expressed in multiple cancers than in the corresponding normal tissues. CPTAC proteomic data also revealed lower total protein expression of EPHX2 in BRCA, ovarian cancer, colon cancer, ccRCC, UCEC, and LUAD (Figure 1C). Moreover, protein expression summary data from the HPA database showed that several cases of prostate and hepatocellular carcinoma and renal cancer displayed moderate cytoplasmic positivity, whereas the other cancer tissues were negative or weakly stained (Figure S2). Meanwhile, we investigated the correlation between EPHX2 expression and tumor pathological stages using the GEPIA2 tool. We observed the effect of EPHX2 expression on tumor stages in COAD, KICH, KIRC, KIRP, LIHC, and PAAD (Figure 1D, all *p* < 0.05), but not in others.

**FIGURE 2:**
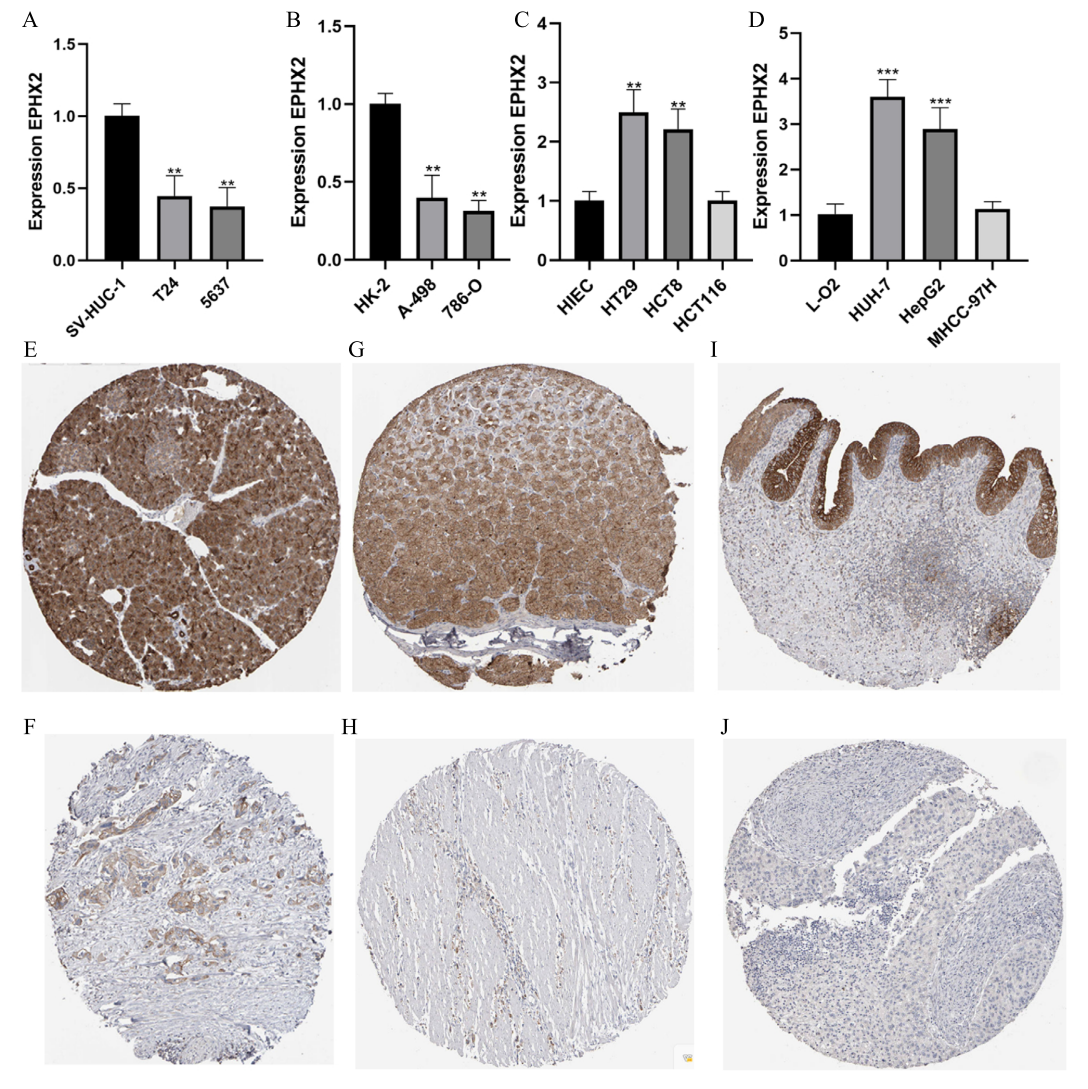
mRNA and protein expression of EPHX2. (A) mRNA expression of EPHX2 in bladder cell lines. (B) mRNA expression of EPHX2 in renal cell lines. (C) mRNA expression of EPHX2 in colon cell lines. (D) mRNA expression of EPHX2 in liver cell lines. (E) Immunohistochemical of normal pancreas. (F) Immunohistochemical of PAAD. (G) Immunohistochemical of stomach tissue. (H) Immunohistochemical of STAD. (I) Immunohistochemical of normal urinary bladder. (J) Immunohistochemical of BLCA. **p < 0.01; ***p < 0.001.

Furthermore, we experimentally validated EPHX2 mRNA expression differences between cancer and normal cell lines. Our results indicated that EPHX2 expression was significantly lower in human bladder cancer cell lines (T24 and 5637) compared with the human ureter epithelial cell line (SV‐HUC‐1) (Figure 2A). Furthermore, EPHX2 expression was low in two renal cancer cell lines, A‐498 and 786‐O, relative to the human renal proximal convoluted tubular epithelial cell line (HK‐2) (Figure 2B). Additionally, the expression of EPHX2 in the human colon carcinoma cell lines, HT29 and HCT8, was significantly increased compared with the human intestinal epithelial cell line (HIEC) (Figure 2C). Compared with the normal human hepatocyte cell line L‐O2, EPHX2 was highly expressed in the human hepatoma cell lines, HUH‐7 and HepG2 (Figure 2D). Immunohistochemistry results from the HPA database demonstrated that EPHX2 was similarly expressed in most cancer types compared to normal tissues, aligning with mRNA expression profiles in the TCGA dataset, such as in PAAD, STAD, and BLCA(Figure 2E–J). These findings suggest that EPHX2 may function as a tumor suppressor in numerous types of tumors, and its clinical significance warrants further exploration.

### 3.2 The Potential Prognostic and Diagnostic Value of EPHX2 Expression in Various Cancers

**FIGURE 3:**
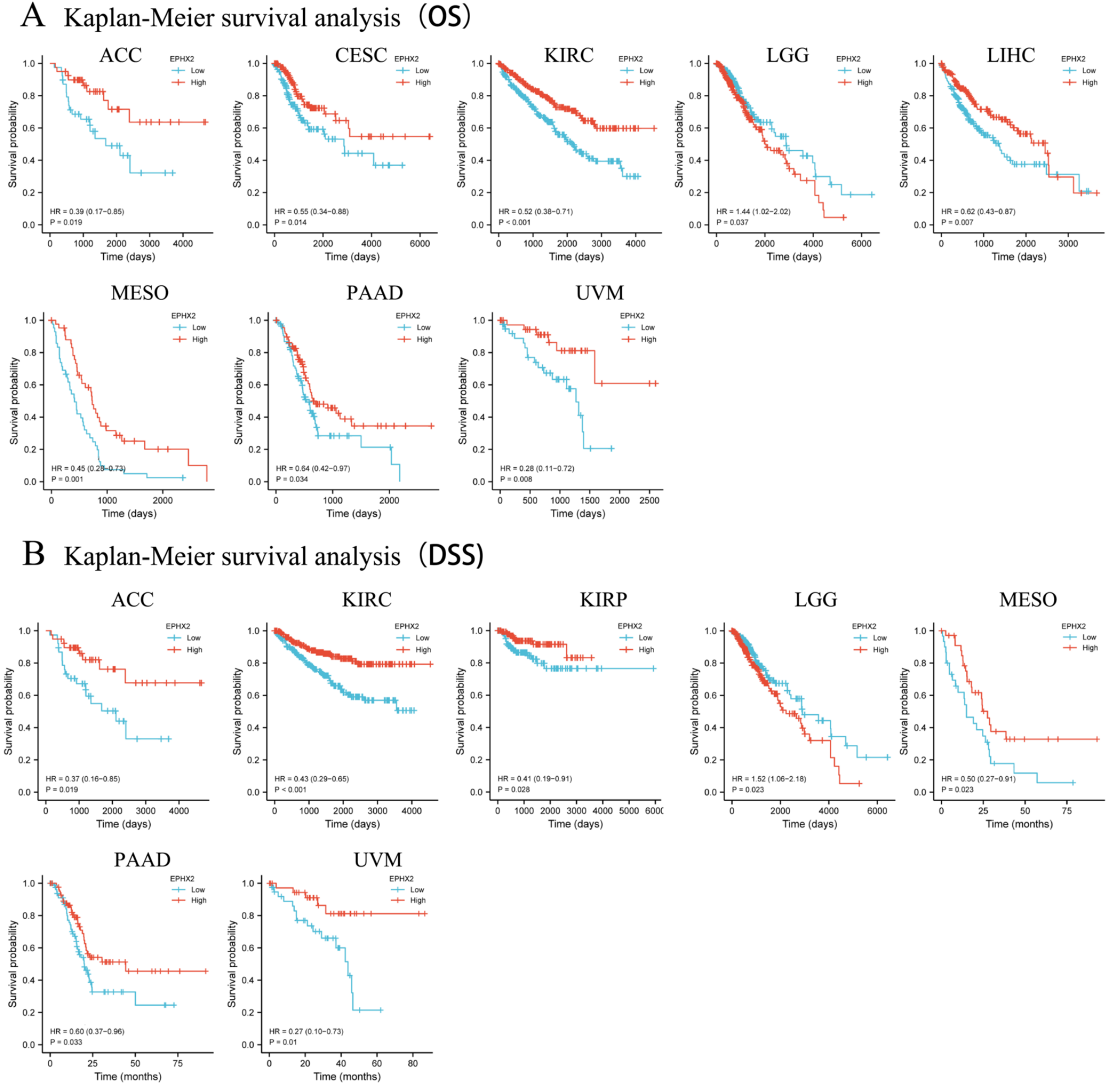
Correlation of EPHX2 expression and pan‐cancer prognosis. (A) Pan‐cancer KM OS analyses of EPHX2 expression in indicated tumor types from the TCGA database. (B) Pan‐cancer KM DSS analyses of EPHX2 expression in indicated tumor types from the TCGA database. The median value of EPHX2 in each tumor was taken as the cut‐off value. DSS, disease‐specific survival; OS, overall survival.

**FIGURE 4:**
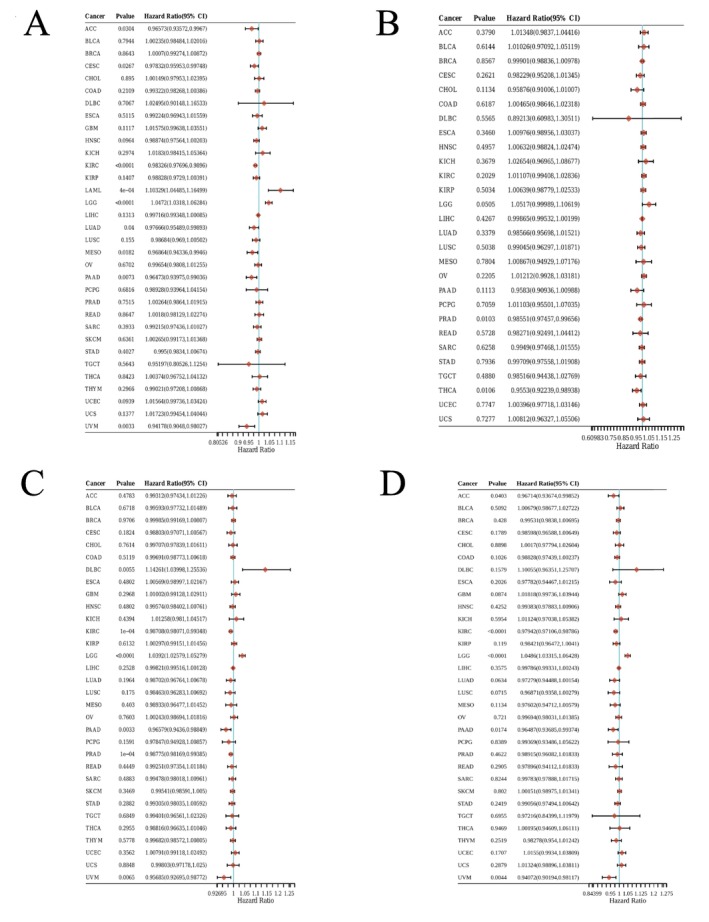
Prognostic significance of EPHX2 for OS, DSS, DFS, and PFS of patients. The univariate Cox regression analysis results of EPHX2 expression in pan‐cancer for OS (A), DSS (B), DFS (C), and PFS (D) of patients. DFS, disease‐free survival; DSS, disease‐specific survival; OS, overall survival; PFS, progression‐free survival.

**FIGURE 5:**
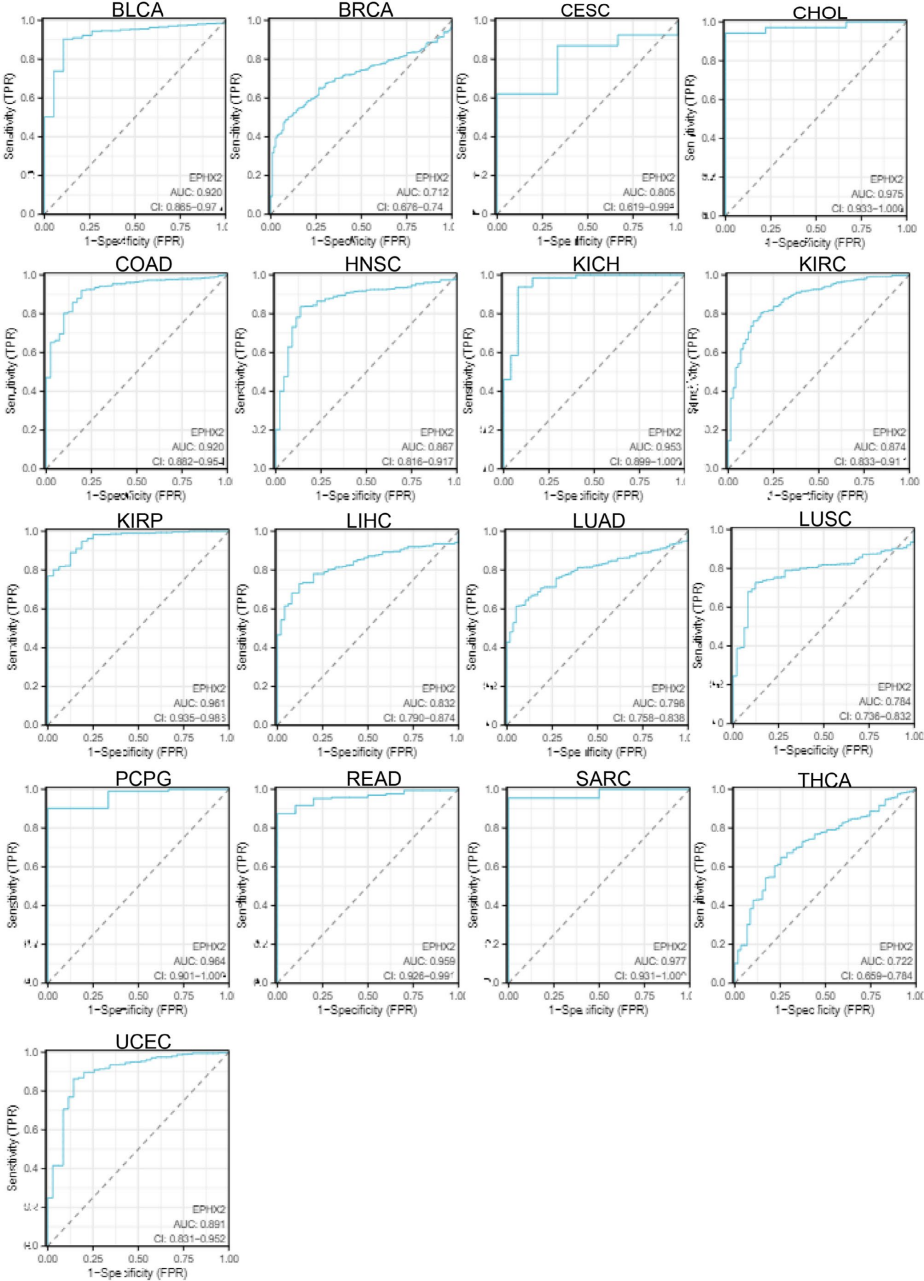
Correlation of EPHX2 expression and pan‐cancer diagnosis in TCGA database. Diagnostic value of EPHX2 expression in pan‐cancer, as determined by ROC curve analysis. AUC, area under the ROC curve; ROC, receiver operating characteristic curve.

Subsequently, we investigated the prognostic significance of EPHX2 in cancer patients by utilizing several databases. The results of KM OS analysis demonstrated that EPHX2 was identified as a protective factor for patients with ACC, CESC, KIRC, LIHC, Mesothelioma (MESO), PAAD, and uveal melanoma (UVM), and as a risk factor for patients with LGG (Figure 3A). Similarly, the KM DSS analysis revealed that EPHX2 acted as a protective factor for patients with ACC, KIRC, KIRP, MESO, PAAD, and UVM, and as a risk factor for patients with LGG (Figure 3B). Moreover, Cox OS analysis suggested that EPHX2 was a protective factor in ACC, CESC, KIRC, LUAD, MESO, PAAD, and UVM, but a risk factor in acute myeloid leukemia (LAML) and LGG (Figure 4A). DSS analysis showed that EPHX2 was a favorable prognostic factor in PRAD and THCA (Figure 4B). For DFS, EPHX2 was more likely to be a favorable factor in KIRC, PAAD, PRAD, and UVM, but deleterious in lymphoid neoplasm diffuse large B‐cell lymphoma (DLBC) and LGG (Figure 4C). PFS analysis revealed that EPHX2 was a risk factor in LGG, but not in ACC, KIRC, PAAD, or UVM (Figure 4D). Furthermore, PrognoScan GEO datasets revealed that EPHX2 was oncogenic in blood cancer and protective in colorectal, eye, lung, prostate, and soft tissue cancers (Figure S3). Notably, the precise role of EPHX2 in BRCA was debatable (such as OS of GSE9893, OS of E‐TABM‐158, RFS of E‐TABM‐158, and DSS of E‐TABM‐158: HR > 1, *p* < 0.05, which differed remarkably from other datasets in BRCA: all HR < 1, *p* < 0.05) (Figure S3). These inconsistencies could be due to different data collection methods, sample sizes, and hypothetical mechanisms of different biological characteristics. Figure S3 summarizes the detailed outcomes. These observations propose that EPHX2 may serve as an innovative predictor for the prognosis of individuals with cancer. The diagnostic potential of EPHX2 was evaluated using ROC curves. As shown in Figure 5, EPHX2 demonstrated excellent diagnostic accuracy in BLCA, CHOL, COAD, KICH, KIRP, PCPG, READ, and SARC (AUCs > 0.9), and moderate diagnostic accuracy in BRCA, CESC, HNSC, KIRC, LIHC, LUAD, LUSC, THCA, and UCEC (AUCs between 0.7 and 0.9). Collectively, these data demonstrate that EPHX2 is a highly valuable tumor diagnostic biomarker across a wide range of tumor types, such as in CESC and KIRC.

### 3.3 Correlation of EPHX2 Expression With the Clinical Characteristics of Cancer Patients

Table 1 depicts the correlation of clinical information with EPHX2 expression in KIRC and CESC. EPHX2 expression was linked to the T stage, N stage, M stage, pathologic stage, gender, and race in KIRC, and the age in CESC. Univariate analysis revealed a strong association of OS with the pathologic T stage, pathologic M stage, pathologic stage, age, and EPHX2 in KIRC, and pathologic T stage, pathologic N stage, pathologic M stage, clinical stage, and EPHX2 in CESC (Table 2; *p* < 0.05). Multivariate analysis, considering these clinicopathological characteristics, further indicated a direct correlation between EPHX2 and OS in CESC (Table 2; *p* < 0.05).

**TABLE 1:** Correlation of the EPHX2 expression and the clinical characteristics of patients with kidney renal clear cell carcinoma (KIRC) and cervical squamous cell carcinoma and endocervical adenocarcinoma (CESC).

KIRC					CESC				
Characteristics	Low	High	*p*	Method	Characteristics	Low	High	*p*	Method
*n*	270	271			*n*	153	153		
Pathologic T stage, *n* (%)			**< 0.001**	Chisq test	Pathologic T stage, *n* (%)			0.848	Yates' correction
T1	115 (21.3%)	164 (30.3%)			T1	67 (27.6%)	73 (30%)		
T2	32 (5.9%)	39 (7.2%)			T2	38 (15.6%)	34 (14%)		
T3	114 (21.1%)	66 (12.2%)			T3	9 (3.7%)	12 (4.9%)		
T4	9 (1.7%)	2 (0.4%)			T4	5 (2.1%)	5 (2.1%)		
Pathologic N stage, *n* (%)			**0.012**	Chisq test	Pathologic N stage, *n* (%)			0.12	Chisq test
N0	118 (45.7%)	124 (48.1%)			N0	71 (36.4%)	63 (32.3%)		
N1	13 (5%)	3 (1.2%)			N1	25 (12.8%)	36 (18.5%)		
Pathologic M stage, *n* (%)			**0.026**	Chisq test	Pathologic M stage, *n* (%)			0.485	Chisq test
M0	213 (41.9%)	216 (42.5%)			M0	55 (21.5%)	61 (23.8%)		
M1	50 (9.8%)	29 (5.7%)			M1	7 (2.7%)	4 (1.6%)		
					MX	68 (26.6%)	61 (23.8%)		
Pathologic stage, *n* (%)			**< 0.001**	Chisq test	Clinical stage, *n* (%)			0.755	Chisq test
Stage I	111 (20.6%)	162 (30.1%)			Stage I	83 (27.8%)	79 (26.4%)		
Stage II	27 (5%)	32 (5.9%)			Stage II	36 (12%)	33 (11%)		
Stage III	79 (14.7%)	44 (8.2%)			Stage III	20 (6.7%)	26 (8.7%)		
Stage IV	51 (9.5%)	32 (5.9%)			Stage IV	10 (3.3%)	12 (4%)		
Gender, *n* (%)			**< 0.001**	Chisq test	Gender, *n* (%)			/	/
Female	73 (13.5%)	114 (21.1%)			Female	/	/		
Male	197 (36.4%)	157 (29%)			Male	/	/		
Race, *n* (%)			**< 0.001**	Yates' correction	Race, *n* (%)			0.573	Chisq test
Asian	4 (0.7%)	4 (0.7%)			Asian	9 (3.4%)	11 (4.2%)		
Black or African American	16 (3%)	41 (7.7%)			Black or African American	14 (5.4%)	17 (6.5%)		
White	247 (46.3%)	222 (41.6%)			White	112 (42.9%)	98 (37.5%)		
Age, *n* (%)			0.576	Chisq test	Age, *n* (%)			**< 0.001**	Chisq test
≤ 60	131 (24.2%)	138 (25.5%)			≤ 50	109 (35.6%)	79 (25.8%)		
> 60	139 (25.7%)	133 (24.6%)			> 50	44 (14.4%)	74 (24.2%)		

*Note:* The meaning of the bold values was regarded as statistically significant. The comparison of baseline data between groups was performed using the Chi‐square test when all expected frequencies exceeded 5 and the total sample size was 40 or greater. When expected frequencies ranged from 1 to less than 5, with a total sample size of 40 or greater, the Chi‐square test with Yates' correction was applied.

**TABLE 2:** Univariate and multivariate analyses of overall survival.

Cancer type	Characteristics	Total (*N*)	Univariate analysis		Multivariate analysis	
			Hazard ratio (95% CI)	*p*	Hazard ratio (95% CI)	*p*
KIRC	Pathologic T stage	541		**< 0.001**		
	T1	279	Reference		Reference	
	T2	71	1.490 (0.895–2.481)	0.125	0.221 (0.069–0.710)	**0.011**
	T3 and T4	191	3.555 (2.536–4.982)	**< 0.001**	0.322 (0.129–0.800)	**0.015**
	Pathologic M stage	508		**< 0.001**		
	M0	429	Reference		Reference	
	M1	79	4.401 (3.226–6.002)	**< 0.001**	0.830 (0.200–3.435)	0.797
	Pathologic stage	538		**< 0.001**		
	Stage I	273	Reference		Reference	
	Stage II	59	1.183 (0.638–2.193)	0.594	5.539 (1.478–20.757)	**0.011**
	Stage III	123	2.649 (1.767–3.971)	**< 0.001**	7.467 (2.853–19.539)	**< 0.001**
	Stage IV	83	6.622 (4.535–9.670)	**< 0.001**	24.452 (4.474–133.634)	**< 0.001**
	Age	541		**< 0.001**		
	≤ 60	269	Reference		Reference	
	> 60	272	1.791 (1.319–2.432)	**< 0.001**	1.725 (1.261–2.360)	**< 0.001**
	EPHX2	541				
	Low	270	Reference		Reference	
	High	271	0.472 (0.346–0.646)	**< 0.001**	0.636 (0.405–1.001)	0.050
CESC	Pathologic T stage	243		**< 0.001**		
	T1	140	Reference		Reference	
	T2	72	1.140 (0.557–2.333)	0.720	0.344 (0.062–1.910)	0.223
	T3 and T4	31	4.019 (2.072–7.797)	**< 0.001**	6.616 (2.009–21.783)	**0.002**
	Pathologic N stage	195		**0.003**		
	N0	134	Reference		Reference	
	N1	61	2.844 (1.446–5.593)	**0.002**	3.397 (1.644–7.018)	**< 0.001**
	Pathologic M stage	256		**0.048**		
	M0 and M1	127	Reference		Reference	
	MX	129	1.708 (0.998–2.925)	0.051	1.650 (0.801–3.400)	0.174
	Clinical stage	299		**0.004**		
	Stage I	162	Reference		Reference	
	Stage II	69	0.812 (0.413–1.599)	0.547	1.400 (0.211–9.302)	0.728
	Stage III and Stage IV	68	2.248 (1.347–3.749)	**0.002**	0.416 (0.118–1.468)	0.173
	EPHX2	306				
	Low	153	Reference		Reference	
	High	153	0.515 (0.319–0.831)	**0.007**	0.463 (0.224–0.955)	0.037

*Note:* The meaning of the bold values was regarded as statistically significant.

### 3.4 The Genetic Alterations of EPHX2 Across Different Tumor Types

**FIGURE 6:**
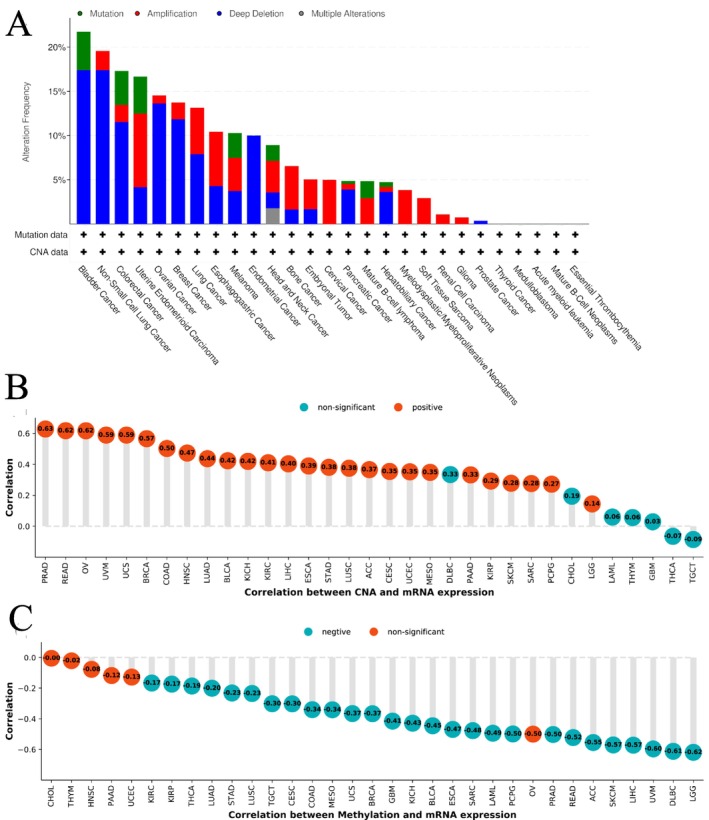
Genetic alteration analysis. (A) The alteration frequency with the mutation type of EPHX2 in TCGA pan‐cancer using the cBioportal database. (B) The correlation between EPHX2 expression and CNA. (C) The correlation between EPHX2 expression and DNA methylation. CNA, copy number alteration.

The assessment of EPHX2 mutations, copy number alteration (CNA), and methylation conditions in pan‐cancer indicated that EPHX2 genomic modifications were higher than 20% in BLCA tumor samples, with “deep deletion” being the most common type (Figure 6A). EPHX2 expression had the strongest positive correlation with CNA in PRAD (*r* = 0.63 *p* < 0.05), READ (*r* = 0.62, *p* < 0.05), and OV (*r* = 0.62, *p* < 0.05) (Figure 6B). Additionally, significant negative correlations were found between EPHX2 expression and DNA methylation in LGG, DLBC, UVM, LIHC, skin cutaneous melanoma (SKCM), ACC, and READ (*r* > 0.50, *p* < 0.05) (Figure 6C). These findings demonstrate that genetic alterations could be related to the transcriptional expression of EPHX2.

### 3.5 Immune Landscape and Characteristics of EPHX2 in the TME

**FIGURE 7:**
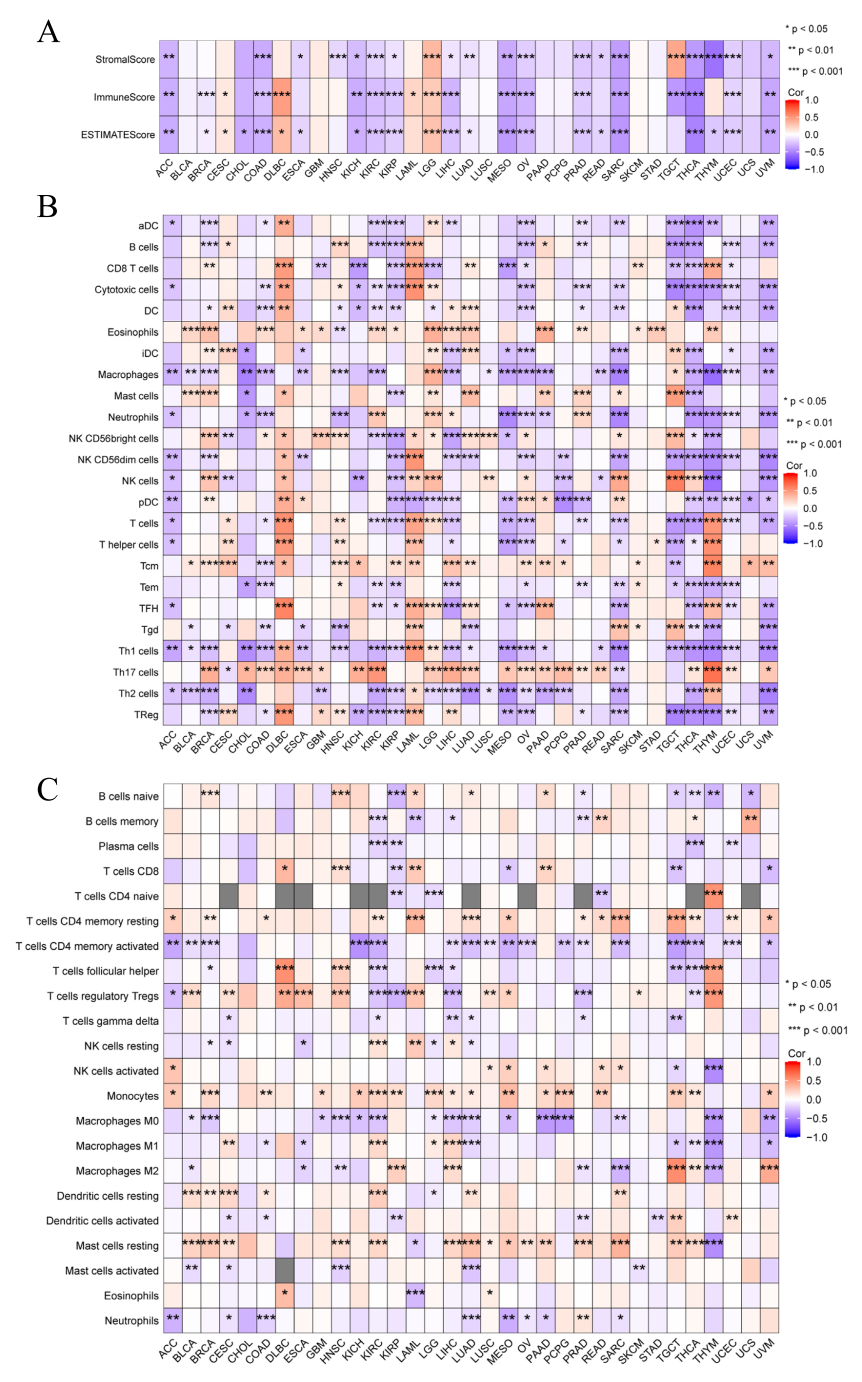
Relationships between EPHX2 expression and TME factors. (A) Correlations of EPHX2 expression with stromal scores, immune scores, and ESTIMATE Scores in pan‐cancer. (B) Relationships between EPHX2 levels and immune infiltrates analyzed by the ssGSEA algorithm. (C) Relationships between EPHX2 levels and immune infiltrates analyzed by the CIBERSORT algorithm. Red indicates a positive correlation, and blue indicates a negative correlation. Darker color indicates stronger correlations. *p < 0.05; **p < 0.01; ***p < 0.001. TME, tumor microenvironment.

The altered immune profile in the TME has recently been recognized as significantly impacting carcinogenesis and prognosis [23, 24]. The ESTIMATE algorithm [19] was applied to determine the immune and stroma scores for each sample in the TCGA database to evaluate the immunological features of EPHX2 in the TME from different cancer types. We discovered that EPHX2 expression was significantly negatively associated with immune scores in ACC, BRCA, COAD, KICH, KIRC, KIRP, LIHC, MESO, OV, PRAD, SARC, TGCT, THCA, UCEC, and UVM, while a positive association was observed in CESC, DLBC, LAML, and LGG (Figure 7A). Similarly, EPHX2 expression showed a negative correlation with stromal scores for ACC, COAD, ESCA, HNSC, KICH, KIRC, KIRP, LIHC, LUAD, MESO, OV, PRAD, READ, SARC, THCA, THYM, UCEC, and UVM, whereas a positive correlation was observed in LGG and TGCT (Figure 7A). EPHX2 expression was negatively correlated with estimated scores for ACC, BRCA, COAD, KICH, KIRC, KIRP, LIHC, LUAD, MESO, OV, PRAD, READ, SARC, THCA, THYM, UCEC, and UVM, but positively correlated with estimated scores in DLBC and LGG (Figure 7A). These data demonstrated that EPHX2 repression was strongly associated with high immune infiltrations in some tumor types.

**FIGURE 8:**
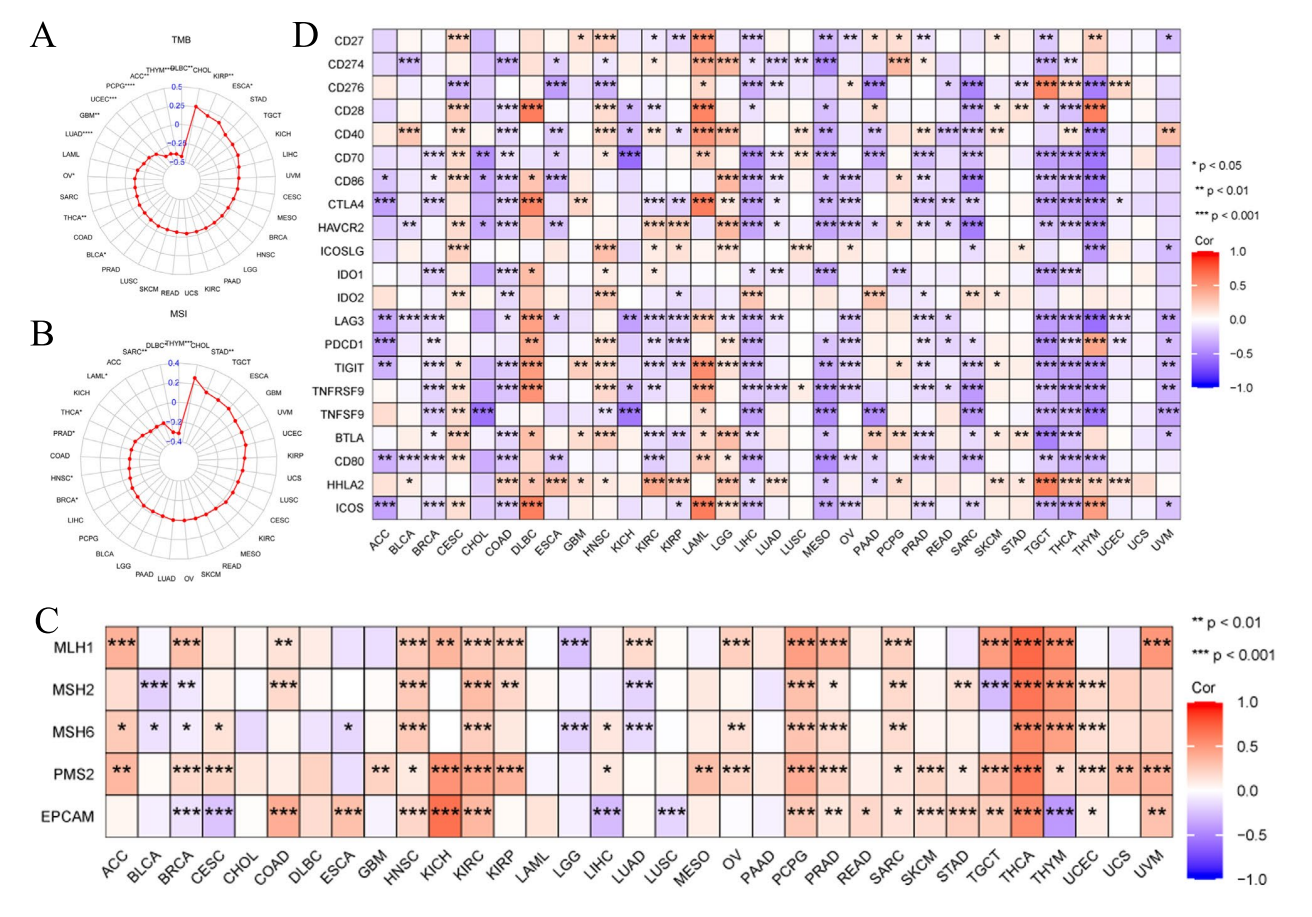
Relationships between EPHX2 expression and TMB, MSI, MMR genes, and ICPs in pan‐cancer. (A) Relationships between EPHX2 expression and TMB displayed by the radar chart. (B) Relationships between EPHX2 expression and MSI displayed by the radar chart. (C) Relationships between EPHX2 expression and MMR gene expression in 33 cancer types. (D) Relationships between EPHX2 expression and ICPs in pan‐cancer. Red indicates a positive correlation, and blue indicates a negative correlation. Darker color indicates stronger correlations. *p < 0.05; **p < 0.01; ***p < 0.001. ICPs, immune checkpoint genes; MMR, mismatch repair; MSI, microsatellite instability; TMB, tumour mutational burden.

For immune cell infiltrations, ssGSEA and CIBERSORT algorithms were used to quantify immune cells in the TME [25]. The findings revealed that the expression of EPHX2 remained closely associated with the infiltration of Th17 cells, Th1 cells, NK CD56dim cells, NK CD56bright cells, neutrophils, macrophages, T cells CD4 memory activated, mast cells resting, monocytes, and T cells regulatory Tregs in most of the TME (Figure 7B,C). Additionally, we found that EPHX2 expression tended to exhibit positive correlations with TMB in KIRP and ESCA, but inverse correlations in BLCA, THCA, OV, LUAD, GBM, UCEC, PCPG, ACC, THYM, and DLBC (Figures 8A and S4; *p* < 0.05). MSI was negatively associated with BRCA, HNSC, PRAD, THCA, LAML, SARC, DLBC, and THYM, whereas STAD was positively associated with MSI (Figures 8B and S5; *p* < 0.05). As such, the association between EPHX2 expression and MMR genes was further investigated. EPHX2 expression correlated significantly and strongly with MMR gene expression in all 33 cancer types (excluding CHOL, DLBC, LAML, and PAAD). MLH1 and PMS2 were positively associated with EPHX2 in the majority of tumors (Figure 8C). Subsequently, a correlation analysis of EPHX2 expression with ICPs in pan‐cancer was performed, revealing that most ICPs were tightly linked to EPHX2 expression, particularly in BRCA, CESC, COAD, DLBC, KICH, KIRC, KIRP, LAML, LGG, LIHC, MESO, OV, PAAD, PRAD, SARC, TGCT, THCA, THYM, and UVM (Figure 8D). Moreover, the correlation between EPHX2 expression and immunomodulators was investigated using the TISIDB database (*p* < 0.01 and |*R*| > 0.5). As depicted in Figure S6, 24 immune inhibitors were analyzed; EPHX2 expression correlated negatively with IL10 and TGFB1 in SARC, TGFB1 in MESO, and LAG3 in UVM. The correlation analysis of 45 immune stimulators (Figure S7) revealed a negative correlation of EPHX2 expression with ULBP1 and TNFRSF8 in UVM, CD276 in PAAD, and CD276 in SARC. Strikingly, as illustrated in Figure S8, a significant negative correlation was found between EPHX2 expression and that for B2M, HLA‐DOB, and TAP1. These data strongly demonstrate that EPHX2 is a critical component of immune infiltrates in human cancers and holds great promise as a new immunotherapy target in tumor management.

### 3.6 Immunotherapy Response Prediction of EPHX2

**FIGURE 9:**
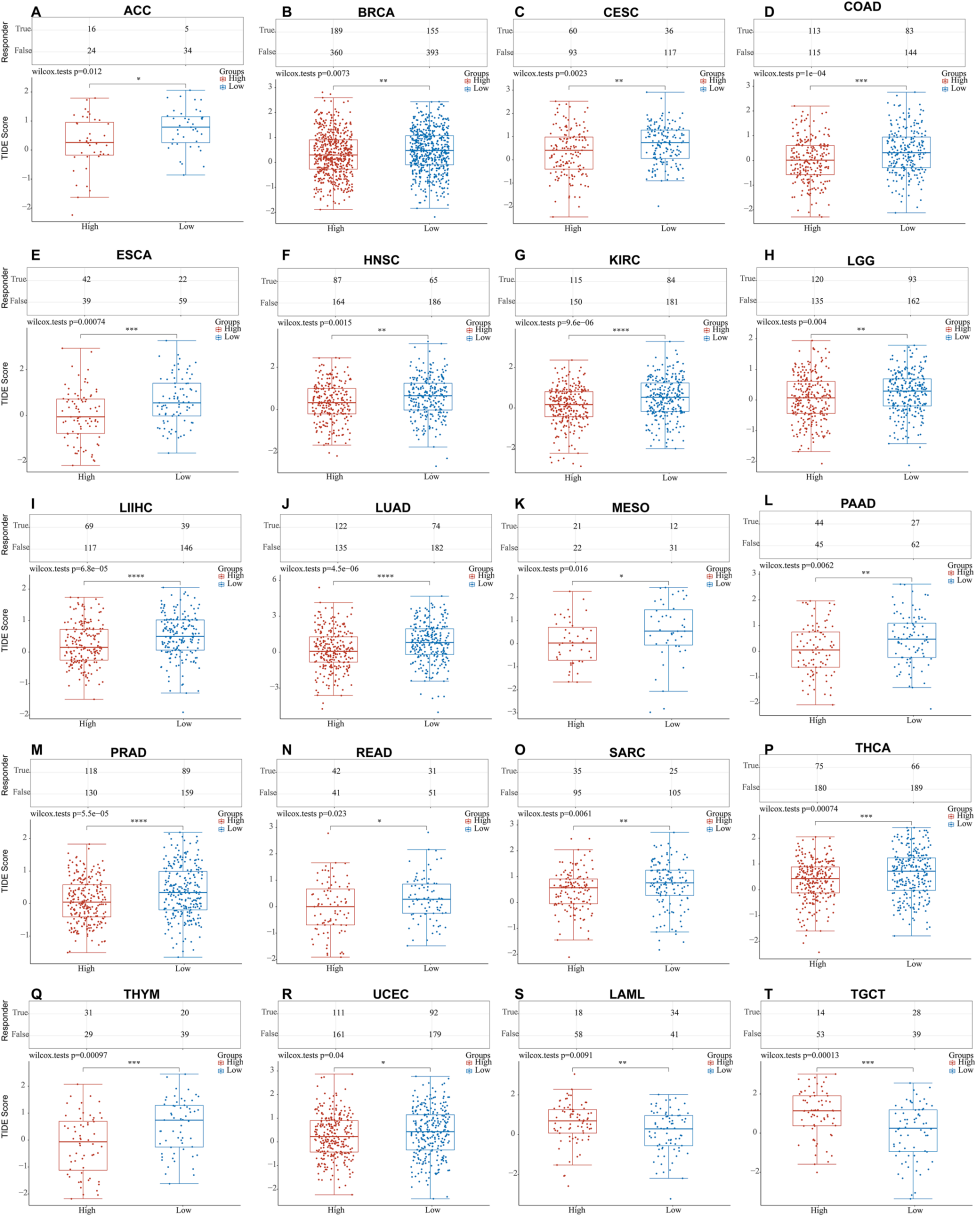
The TIDE score of EPHX2 expression in TCGA tumors. (A–T) Above: Statistical table of immune response of samples in different groups in the prediction results; Below: The distribution of immune response scores in different groups in the prediction results. Red represents high expression group; blue represents low expression group (*p < 0.05; **p < 0.01; ***p < 0.001).

ICB has revolutionized cancer therapy and could provide comprehensive and long‐lasting responses [26, 27]. We used the TIDE algorithm to predict the ICB responses [28] of high and low EPHX2 expression groups based on TCGA expression profile data. Patients with high EPHX2 expression in 18 TCGA tumor types (ACC, BRCA, CESC, COAD, ESCA, HNSC, KIRC, LGG, LIHC, LUAD, MESO, PAAD, PRAD, READ, SARC, THCA, THYM, and UCEC) achieved lower TIDE scores compared with the low EPHX2 group (Figure 9). However, in LAML and TGCT, the results were opposite (Figure 9). Because patients with higher TIDE scores were considerably more likely to have a higher chance of antitumor immune escape and a lower response rate to ICB treatment [29], patients with low‐risk scores appeared to be more susceptible and sensitive to ICB treatment.

### 3.7 EPHX2 Expression Patterns at Single‐Cell Levels

**FIGURE 10:**
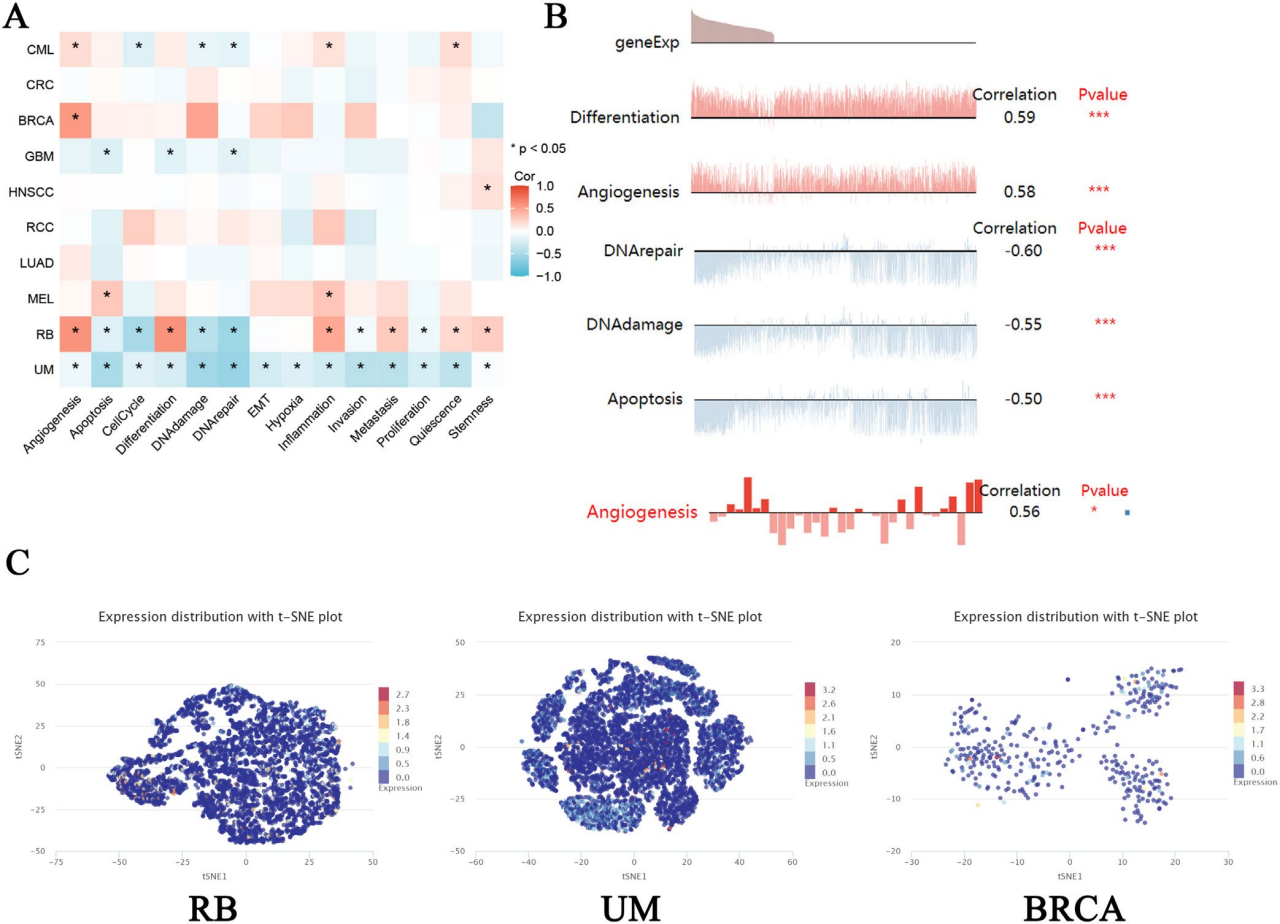
The expression levels of EPHX2 at single‐cell levels. (A, B) The relationships between EPHX2 expression and different functional states in tumors were explored by the CancerSEA tool (*p < 0.05; **p < 0.01; ***p < 0.001). (C) EPHX2 expression profiles were shown at single cells from RB, UM, and BRCA by T‐SNE diagram. T‐SNE describes the distribution of cells; every point represents a single cell, and the color of the point represents the expression level of the gene (gene list) in the cell.

Single‐cell transcriptome sequencing has emerged as a critical tool for analyzing candidate molecules for functional and phenotypic analysis at a single‐cell level [29, 30, 31]. To further investigate the latent role of EPHX2 in tumors, we analyzed the function of EPHX2 at the single‐cell level using CancerSEA. The findings displayed that, in retinoblastoma (RB), EPHX2 expression had a positive relationship with angiogenesis, differentiation, inflammation, metastasis, quiescence, and stemness. Contrarily, it was negatively linked to apoptosis, cell cycle, DNA damage, and DNA repair(Figure 10A). In UVM, EPHX2 expression showed a negative correlation with apoptosis, DNA damage, DNA repair, invasion, metastasis, and quiescence. This suggests that EPHX2 expression may play a role in tumor progression and resistance to cell death (Figure 10A). In BRCA, EPHX2 expression was positively correlated with angiogenesis, indicating a potential role in promoting vascularization within tumors (Figure 10A). As depicted in Figure 10B, EPHX2 expression was significantly associated with differentiation and angiogenesis in RB, DNA repair, DNA damage, and apoptosis in UVM, and angiogenesis in BRCA. T‐SNE diagrams depicted EPHX2 expression profiles from RB, UVM, and BRCA at the single‐cell level (Figure 10C).

### 3.8 PPI Network of EPHX2 and GSVA Analysis in Pan‐Cancer

**FIGURE 11:**
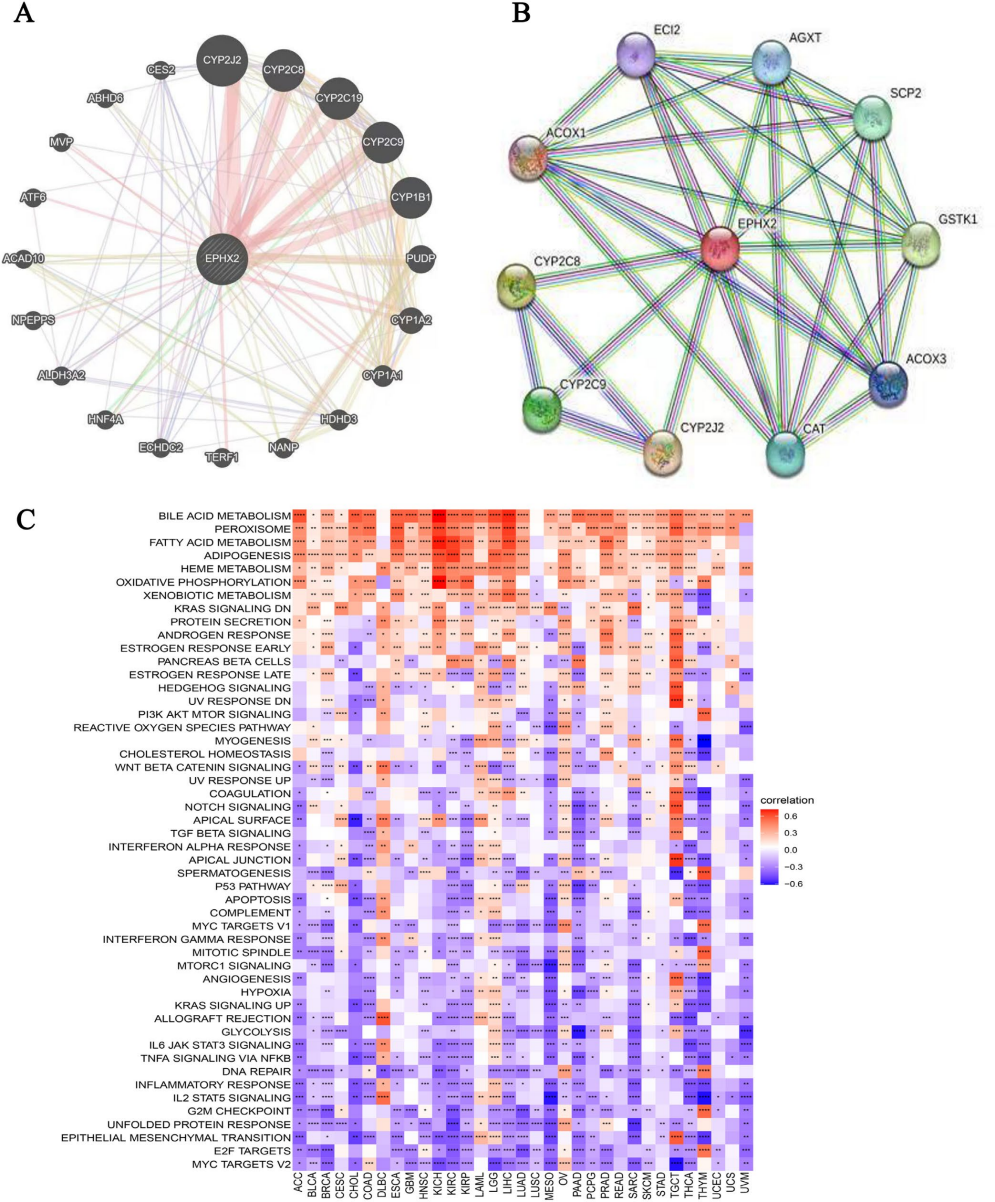
PPI networks and hallmark pathways of EPHX2 in cancers. (A, B) PPI network was constructed using the GeneMANIA and STRING databases. (C) Heatmap showing the enrichment of significant hallmarks sets. Red indicates a positive correlation, and blue indicates a negative correlation. Darker color indicates stronger correlations (Spearman correlation, *p* < 0.05 was considered significant, *p < 0.05, **p < 0.01, ***p < 0.001, and ****p < 0.0001).

Furthermore, the PPI network was constructed using the GeneMANIA and STRING databases, which revealed three genes (including CYP2J2, CYP2C9, and CYP3A4) that were closely related to EPHX2 (Figure 11A,B). Following that, the GSVA analysis results demonstrated that EPHX2 could influence the occurrence and development of diseases such as cancers via multiple distinct signaling pathways. These results strongly demonstrate that EPHX2 expression is positively associated with several metabolism‐related pathways, including bile acid metabolism, peroxisome, fatty acid metabolism, adipogenesis, and heme metabolism, and inversely associated with numerous common cancer‐related pathways, including MYC targets V2, E2F targets, epithelial‐mesenchymal transition, unfolded protein response, G2M checkpoint, and others (Figure 11C; *p* < 0.05). These findings warrant additional examination.

### 3.9 Sensitive Drugs Targeting EPHX2 and the Predictor Genes

**FIGURE 12:**
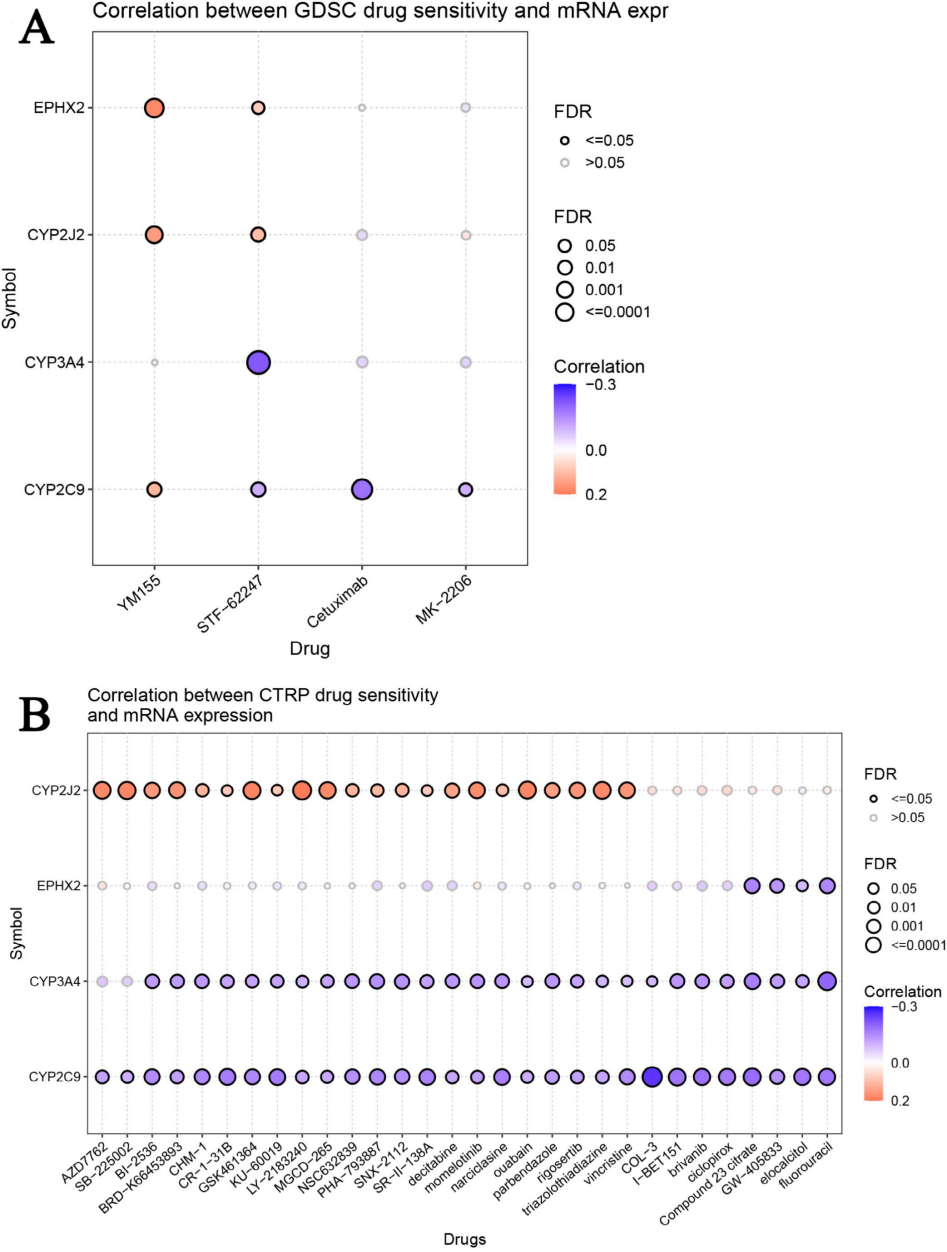
Sensitive drug prediction of EPHX2 and co‐expressed genes in pan‐cancer. Predictive drugs are based on the gene expression in pan‐cancer from the GDSC (A) and CTRP (B) datasets.

We investigated the association between EPHX2 and the expression of co‐expressed genes and patient sensitivity to chemotherapy. Pearson's correlation analysis on the GDSC revealed that drug sensitivity to STF−62247 was linked to the expression of CYP3A4 and CYP2C9 (negative association with IC50). However, drug resistance to YM155 and STF−62247 was associated with the expression of EPHX2 and CYP2J2 (positive association with IC50) (Figure 12A). Pearson's correlation analysis from the CTRP revealed that drug sensitivity to Compound 23 citrate, GW−405 833, elocalcitol, and fluorouracil was associated with the expression of EPHX2, CYP3A4, and CYP2C9 (negative association with IC50). However, drug resistance to AZD7762, SB−225002, BI−2536, BRD−K66453893, CHM−1, CR−1−31B, GSK461364, KU−60019, LY−2183240, MGCD−265, NSC632839, PHA−793887, SNX−2112, SR−II−138A, decitabine, momelotinib, narciclasine, ouabain, parbendazole, rigosertib, triazolothiadiazine, and vincristine was associated with the expression of CYP2J2 (positive association with IC50) (Figure 12B). The results indicate that the dysregulated expression of EPHX2 and the co‐expressed genes potentially play a role in drug resistance to chemotherapy and targeted therapies.

## 4 Discussion

Cancer research has consistently been a prominent focus in biomedical research. Emerging articles have identified links between EPHX2 and clinical disorders [10, 32], particularly neoplastic diseases [33, 34, 35]. We initially undertook a systematic bioinformatic analysis of the molecular landscape of the EPHX2 gene in pan‐cancer using multiple databases to determine its significance in cancer prognosis, progression, staging, diagnosis, and treatment.

Our findings revealed that EPHX2 was generally downregulated in most cancer types according to TIMER, TCGA, and GTEx datasets, implying that EPHX2 may exert a tumor‐suppressive effect. Conversely, qPCR validation in selected cancer cell lines demonstrated an upregulation of EPHX2 in colon carcinoma and hepatoma cell lines compared to normal cells, underscoring the heterogeneity of EPHX2 expression in various cancer contexts and indicating that its functional role may differ depending on the specific tumor type and cellular environment. Furthermore, we found that EPHX2 expression was significantly correlated with tumor stage in COAD, KICH, KIRC, KIRP, LIHC, and PAAD. Recent studies have suggested that EPHX2 deregulation is significantly associated with prostate cancer progression and poor prognosis [36]. Decreased expression of EPHX2 is significantly correlated with LIHC development and may be considered a promising candidate therapeutic target for LIHC [15]. Zhou et al. [14] also confirmed the inhibitory effect of EPHX2 in COAD. We conducted additional qPCR experiments to validate the gene expression levels of EPHX2 in various cancer cell lines, further substantiating its involvement in cancer. This study expanded upon existing knowledge by employing single‐cell sequencing and GSVA analysis to investigate the molecular mechanisms of EPHX2 in greater detail [37, 38]. Our findings indicated that EPHX2 expression is inversely associated with several cancer‐related biological processes, including the cell cycle and apoptosis, thereby impeding tumor growth and progression.

Intriguingly, we found the prognostic value of EPHX2 expression in a variety of tumors by cross‐referencing databases, particularly the consistent prognostic value in CESC, KIRC, KIRP, LIHC, and PAAD. Previous studies have also identified EPHX2 as a protective prognostic factor in oncological outcomes (e.g., PRAD [36] and LIHC [15]). Nonetheless, information on the prognostic value of EPHX2 in other solid tumor types remains scarce [39]. The role of EPHX2 in BRCA was controversial in the PrognoScan database. Therefore, substantially larger sample sizes are required to verify the role of EPHX2 in the different types of BRCA prognosis and survival. In addition, EPHX2 expression had satisfactory diagnostic accuracy for BLCA, CHOL, COAD, KICH, KIRP, PCPG, READ, SARC, BRCA, CESC, HNSC, KIRC, LIHC, LUAD, LUSC, THCA, and UCEC (AUCs greater than 0.7, and even 0.9). Current evidence strongly suggests that EPHX2 is a valuable tumor biomarker in different tumors, such as in CESC and KIRC.

Mutation arises from changes in the gene sequence, influencing tumor development [40]. In contrast, epigenetic alterations do not change the primary DNA sequence [41]. However, both mutations and epigenetic alterations result in abnormal gene expression. Genomic research has revealed that deletion is the most common gene alteration of EPHX2 in most cancers. Moreover, BLCA had the highest alteration numbers, followed by non‐small cell lung cancer, colorectal cancer (CRC), and uterine endometrioid carcinoma. Previous studies have shown that the deletion of the EPHX2 gene decreases the survival rate after cardiac arrest [42]. Genetic variations in EPHX2 have been linked to the occurrence of stroke in rats and humans [43, 44]. EPHX2 encodes a cytosolic epoxide hydrolase, and its depletion in various cancers may induce aberrant expression, resulting in metabolic dysfunctions that could influence cancer progression and metastasis differently depending on the stage of cancer development [45]. Moreover, EPHX2 expression exhibited the strongest positive correlation with CNA in PRAD, READ, and OV, indicating that CNA significantly impacts EPHX2 expression in these cancers. Considering that DNA methylation can silence gene expression [46], we analyzed the relationship of EPHX2 expression with DNA methylation across various cancers. We discovered significant negative correlations between EPHX2 expression and DNA methylation in LGG, DLBC, UVM, LIHC, SKCM, ACC, and READ. Consequently, EPHX2 holds potential as a diagnostic marker for identifying mutations, CNAs, and epigenetic alterations in cancer.

Understanding how the host immune system interacts with tumors is critical for identifying new prognostic biomarkers, developing strategies to decrease drug resistance, and discovering effective targeted therapies [47, 48]. In this report, we systematically addressed the potential role of EPHX2 as a viable immunotherapy target in the TME against cancers. By combining data from various studies on EPHX2 expression in tumor and normal tissues, we uncovered the immunotherapeutic potential of EPHX2 in a range of tumor types. To better understand the potential immune value of EPHX2, we assessed the immune score of cancer patients from the TCGA cohort using the ESTIMATE and CIBERSORT algorithms and discovered that EPHX2 expression was strongly correlated with Th17 cells, Th1 cells, NK CD56dim cells, NK CD56bright cells, neutrophils, macrophages, T cells CD4 memory activated, mast cells resting, monocytes, and T cells regulatory Tregs in most tumors. Furthermore, the associations of EPHX2 with TMB, MSI, MMR genes, and ICPs were investigated across diverse cancer types. Both biomarkers (TMB and MSI) for immunotherapy were significantly associated with EPHX2 in certain cancers. In general, a high TMB value corresponds to the production of more somatic mutation‐related neoantigens [49]. In contrast, MSI is defined as a hypermutator phenotype caused by impaired DNA MMR and is a potential predictive marker for immunotherapy [50]. In DLBC, THYM, and THCA, EPHX2 was inversely correlated with TMB and MSI, suggesting that EPHX2 might indirectly affect the immunotherapy response in these cancers. Emerging evidence has shown that tumor‐infiltrating immune cells play complex and critical roles in the progression and aggressiveness of cancer [51, 52]. The composition of tumor‐infiltrating immune cells influences the immune status of the TME [53, 54]. As key components of the TME [55, 56], tumor‐associated macrophages provide an inflammatory environment that promotes cancer progression [57, 58, 59]. Compelling evidence from clinical studies has demonstrated that high infiltration of tumor‐associated macrophages correlates with poor prognosis [60, 61]. Our results showed that decreased EPHX2 was significantly negatively associated with macrophages for most tumors, but positively associated with macrophages in LGG and TGCT. This suggests that EPHX2‐associated immune infiltration in different tumors might have a complex interplay affecting tumor development and progression. It also demonstrated that changes in EPHX2 expression may alter immune cell infiltration in the TME. Kelly et al. [38] discovered that inhibiting soluble epoxide hydrolase can enhance cancer immunotherapy outcomes, reinforcing our belief that targeting EPHX2‐dependent pathways may be a promising therapeutic strategy.

Additionally, we found that EPHX2 is correlated with immunotherapy response. ICB therapy has emerged as one of the most promising approaches for activating the antitumor immune response, achieving great success in treating various cancers, eliciting durable responses, and prolonging patient survival [62, 63, 64, 65]. Together with previous data, our findings indicate that patients with high EPHX2 expression might be more responsive to ICB in the majority of TCGA tumors. Although this approach has significant therapeutic potential, it is not effective in some patients. Accumulating evidence has indicated that the efficacy of ICB mainly depends on robust anti‐tumor immunity responses, which are commonly considered to be compromised in most tumors [2]. Therefore, these conclusions should be verified in future clinical trials. This study provides new insights into the role of EPHX2 in cancer immunotherapy, revealing associations between EPHX2 and important immunological indicators (immune cell infiltrations, immunomodulators, and immune biomarkers), improving our understanding of the mechanism linking EPHX2 and immunotherapy. These findings underscore the immunological role of EPHX2 in specific cancers, suggesting EPHX2 as an effective target in such cancers, although the association between EPHX2 and TME was not detected in some tumors. Our findings also indicated that EPHX2‐related genes contribute to the occurrence of resistance to small molecule drugs.

This study is limited by inconsistencies in sample size across different datasets, which may impact the strength of our conclusions. Increasing sample size and incorporating more comprehensive sequencing data and clinical information would enhance the reliability of our results. Future studies should elucidate the precise biological pathways and molecular interactions involving EPHX2. Finally, this study primarily utilizes bioinformatics approaches and qRT‐PCR techniques to analyze different cancer cell types. Further experimental validation, including in vitro and in vivo functional studies, is essential to confirm our findings and advance our understanding of EPHX2's role in cancer.

## 5 Conclusions

In summary, EPHX2 was aberrantly expressed in various tumor types and exhibited a strong correlation with clinical progression and prognosis. Our findings suggest that EPHX2 holds potential as a prognostic marker, especially in CESC. Furthermore, the role of EPHX2 in the TME and its impact on the abundance of immune cell infiltrations was significant. EPHX2 may be a valuable biomarker for predicting responses to immunotherapy and guiding individualized immunotherapy strategies for cancer patients.

## Author Contributions

**Weiquan Hu:** funding acquisition (equal); conceptualization (lead); formal analysis (lead); methodology (equal); writing – original draft (lead). **Xiaoli Ding:** funding acquisition (equal); data curation (equal); formal analysis (lead); resources (equal); validation (equal); writing – review and editing (supporting). **Xiangsheng Wu:** funding acquisition (equal); methodology (lead); resources (equal); software (supporting); supervision (supporting); validation (equal); writing – review and editing (supporting). **Xuxiang Xi:** conceptualization (equal); data curation (equal); formal analysis (lead); writing – original draft (lead). **Jing Xu:** conceptualization (equal); data curation (equal); investigation (lead); writing – original draft (supporting). **Shengyun Dai:** methodology (lead); software (supporting); validation (supporting); visualization (supporting). **Jing Chen:** data curation (supporting); investigation (equal); resources (lead); validation (supporting); visualization (supporting). **Suping Hu:** methodology (equal); resources (lead); software (equal). **Qinfei Zhao:** conceptualization (supporting); project administration (lead); writing – original draft (supporting); writing – review and editing (equal). **Fangfang Chen:** funding acquisition (equal); investigation (equal); writing – review and editing (equal).

## Ethics Statement

The authors have nothing to report.

## Conflicts of Interest

The authors declare no conflicts of interest.

## Supporting information


**Figure S1.** The EPHX2 expression levels in tumors and paired adjacent normal tissues in pan‐cancer data of TCGA. Black lines connect paired tissues (*p < 0.05; **p < 0.01; ***p < 0.001). ns, not significant.

**Figure S2.** Pan‐cancer analysis of EPHX2 protein expression level across cancers in the HPA database.

**Figure S3.** Pan‐cancer prognostic analysis of EPHX2 expression in different datasets of cancers in PrognoScan. The red circle represents the HR. HR, hazard ratio.

**Figure S4.** Correlation between EPHX2 gene expression and TMB in TCGA database of KIRP, ESCA, BLCA, THCA, OV, LUAD, GBM, UCEC, PCPG, ACC, THYM, and DLBC. TMB, tumour mutational burden.

**Figure S5.** Correlation between EPHX2 gene expression and MSI in TCGA database of STAD, BRCA, HNSC, PRAD, THCA, LAML, SARC, DLBC, and THYM.

**Figure S6.** The correlation between the EPHX2 expression and immune inhibitors. Red indicates positive correlation and blue indicates negative correlation. The first four strongest associations are shown by dot plots.

**Figure S7.** The correlation between the EPHX2 expression and immune stimulators. Red indicates positive correlation and blue indicates negative correlation. The first four strongest associations are shown by dot plots.

**Figure S8.** The correlation between the EPHX2 expression and MHC molecules. Red indicates positive correlation and blue indicates negative correlation. The first three strongest associations are shown by dot plots.

## Data Availability

Data presented in this study are included in the article/Supporting Information. Further data can be accessed by emailing the authors.
