# Supplementary figures and images for: A Comprehensive Analysis of Epoxide Hydrolase 2 (EPHX2) in Pan‐Cancer
Source: Cancer Rep (Hoboken). 2025 Mar 24;8(3):e70188. doi: 10.1002/cnr2.70188 (PMC11932960; doi:10.1002/cnr2.70188)

The expression of EPHX2  
 $\text{Log}_2(\text{TPM}+1)$

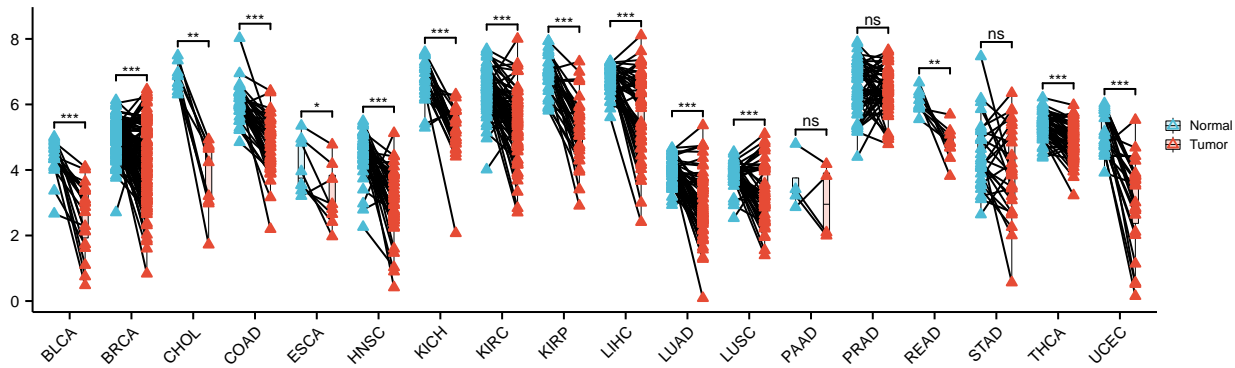

Supplement: Supplementary file 1 — Figure S1. The EPHX2 expression levels in tumors and paired adjacent normal tissues in pan‐cancer data of TCGA. Black lines connect paired tissues (*p < 0.05; **p < 0.01; ***p < 0.001). ns, not significant. [file CNR2-8-e70188-s006.pdf]

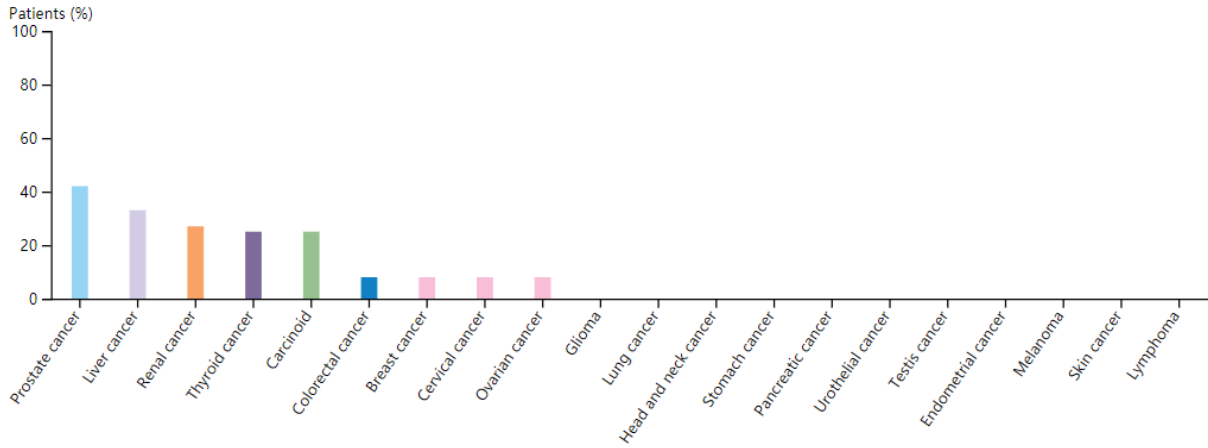

Supplement: Supplementary file 2 — Figure S2. Pan‐cancer analysis of EPHX2 protein expression level across cancers in the HPA database. [file CNR2-8-e70188-s005.pdf]

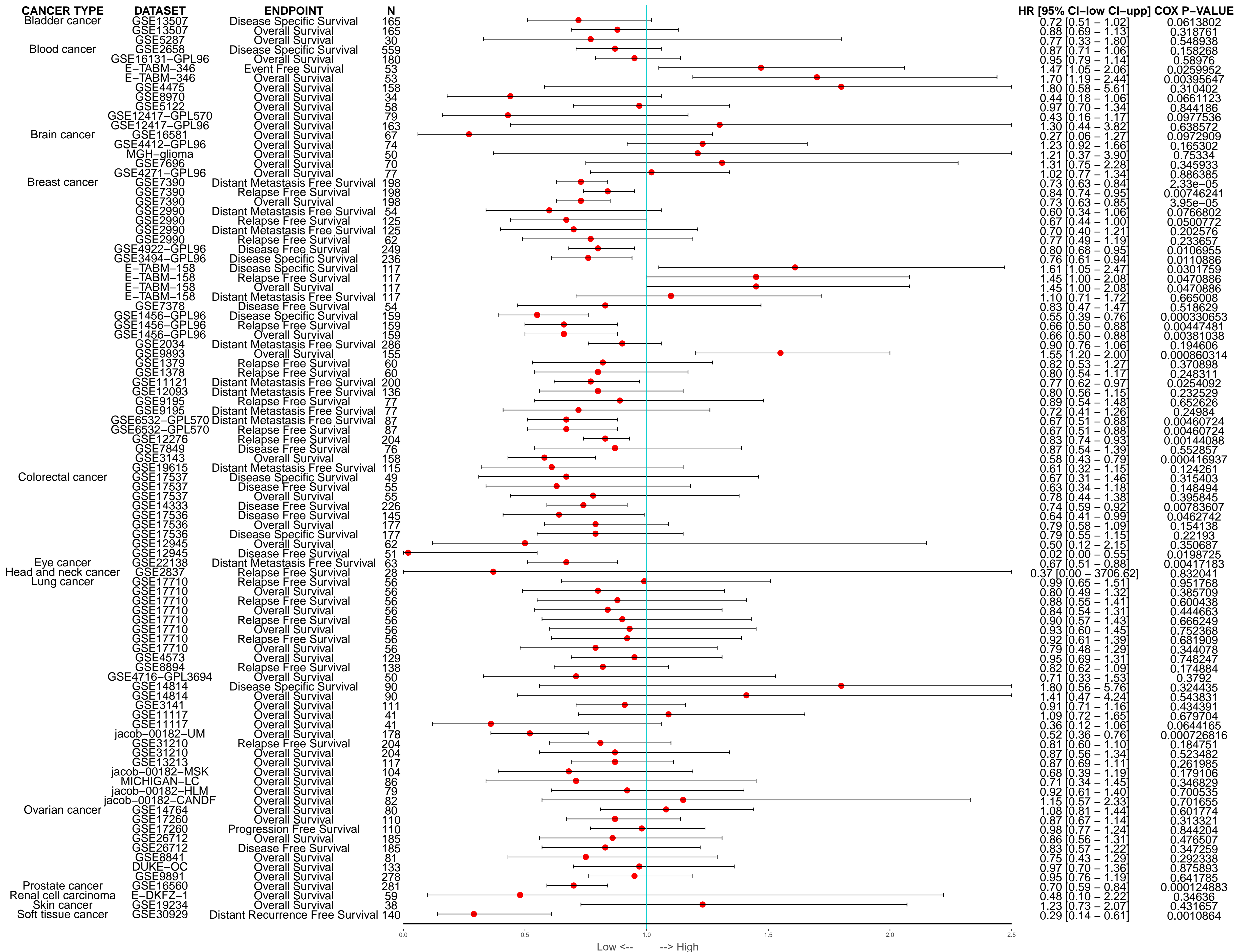

Supplement: Supplementary file 3 — Figure S3. Pan‐cancer prognostic analysis of EPHX2 expression in different datasets of cancers in PrognoScan. The red circle represents the HR. HR, hazard ratio. [file CNR2-8-e70188-s004.pdf]

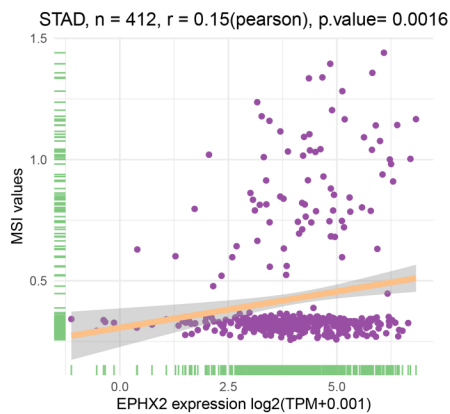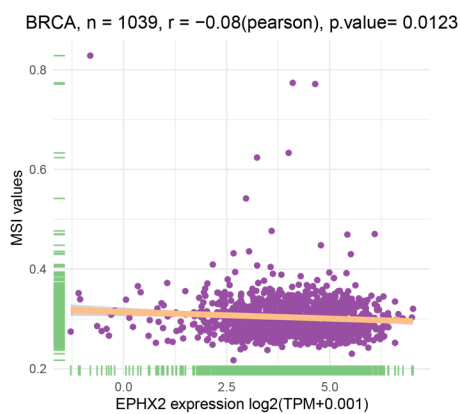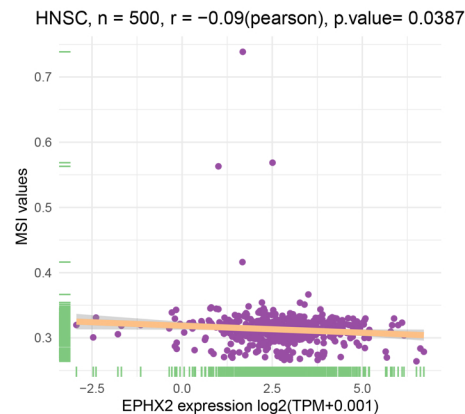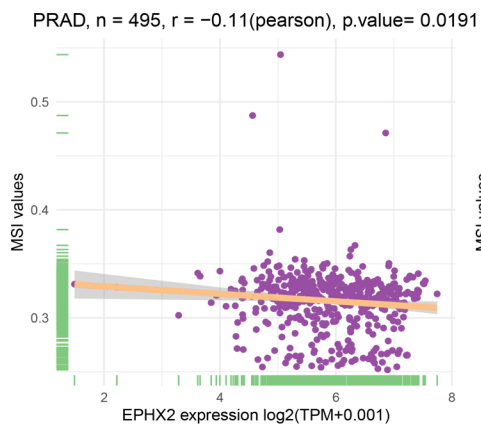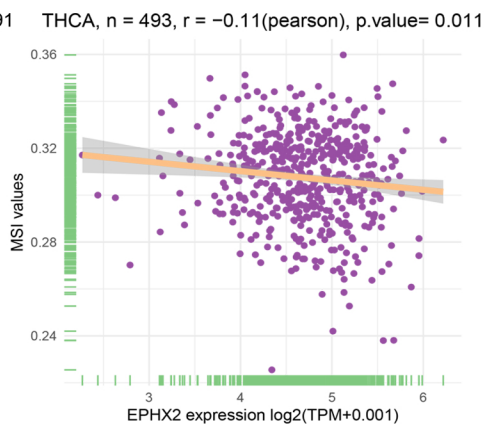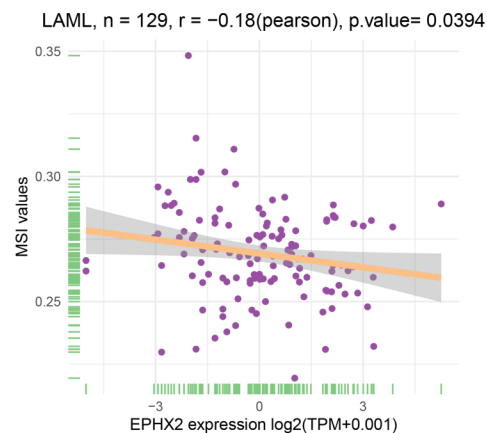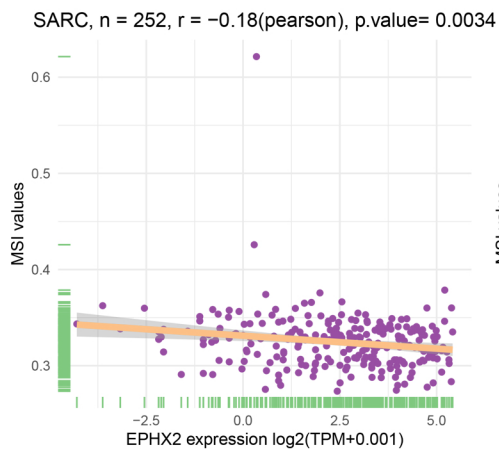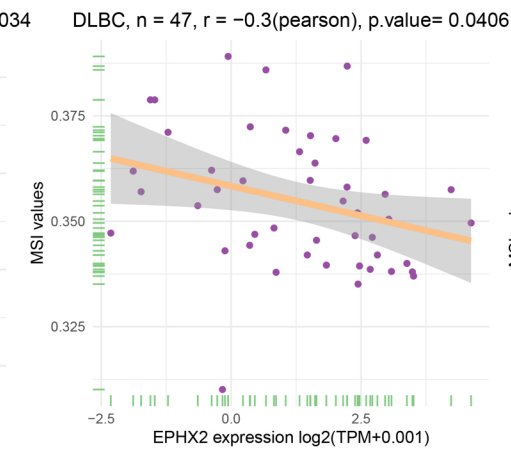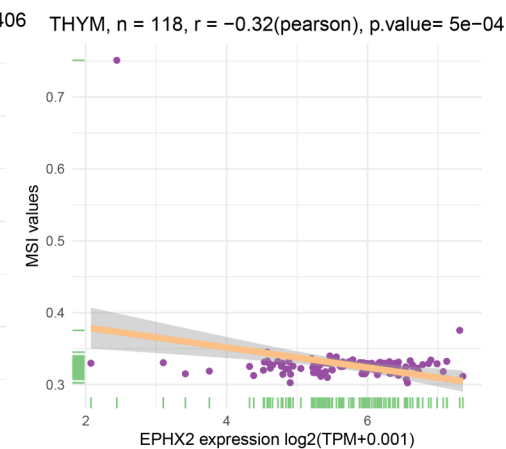

Supplement: Supplementary file 5 — Figure S5. Correlation between EPHX2 gene expression and MSI in TCGA database of STAD, BRCA, HNSC, PRAD, THCA, LAML, SARC, DLBC, and THYM. [file CNR2-8-e70188-s007.pdf]

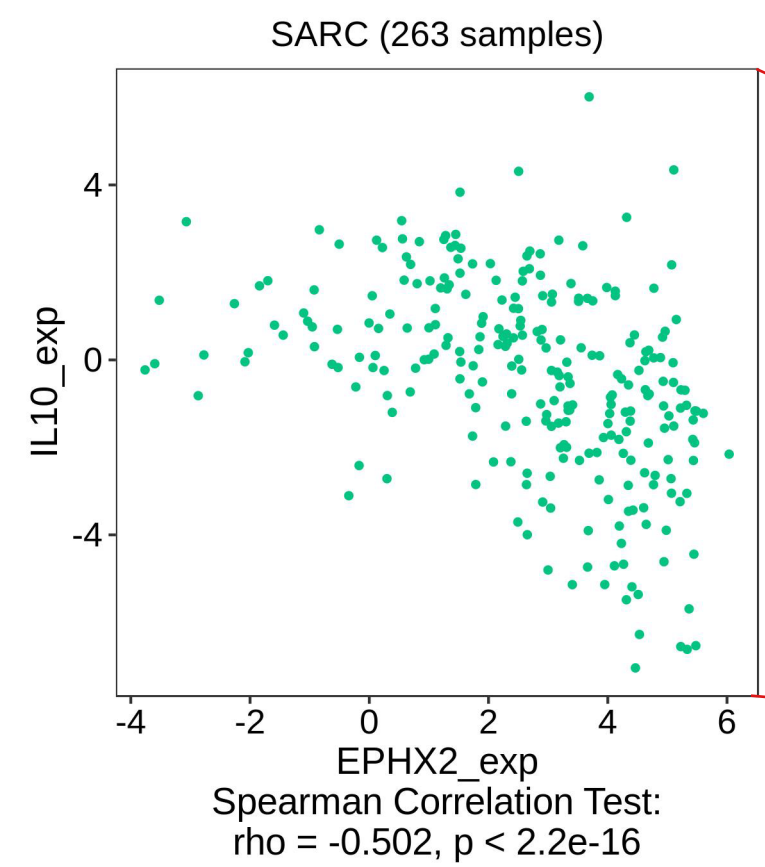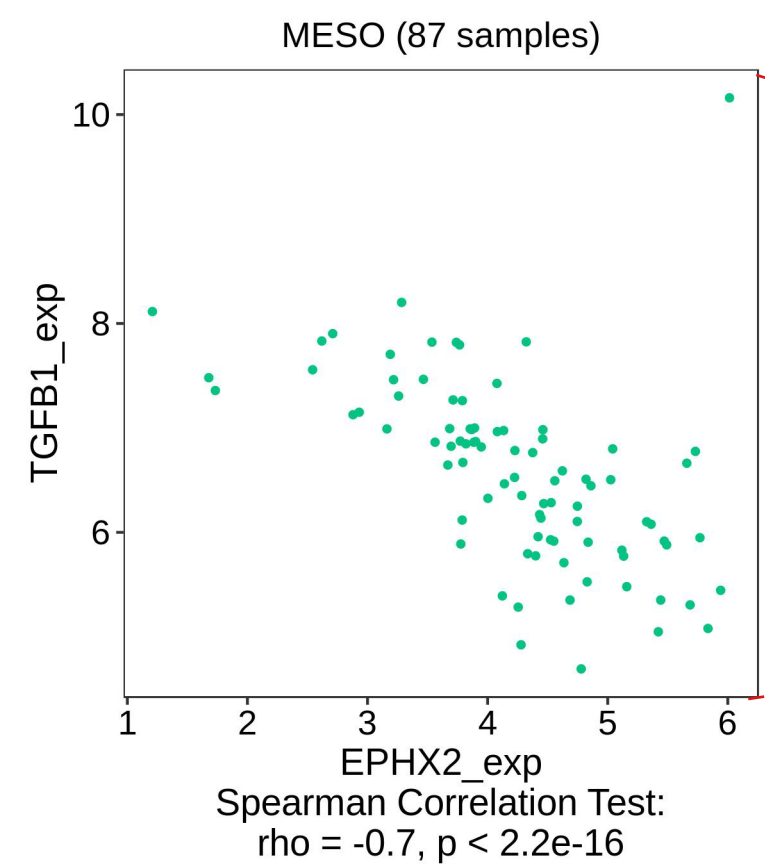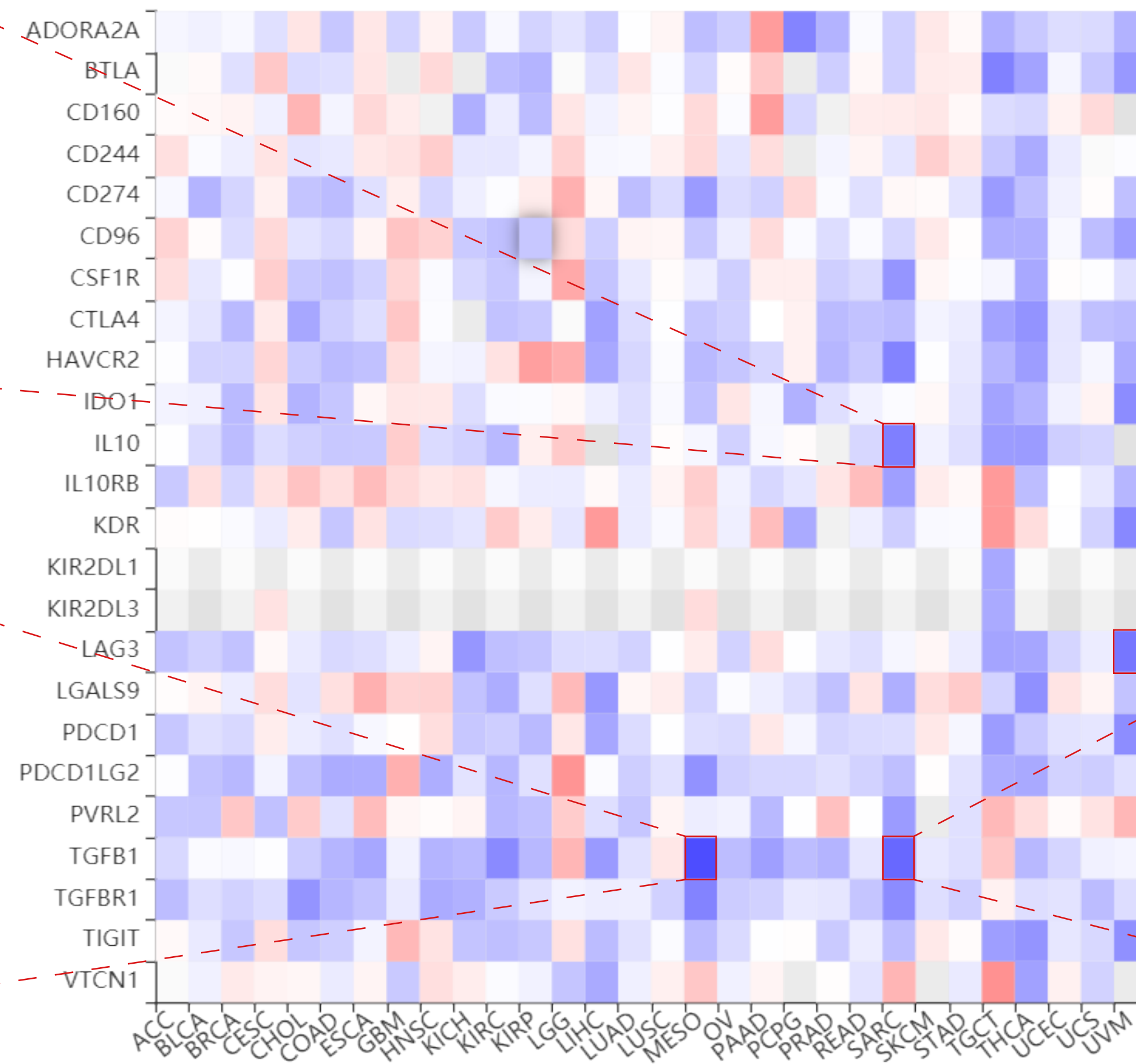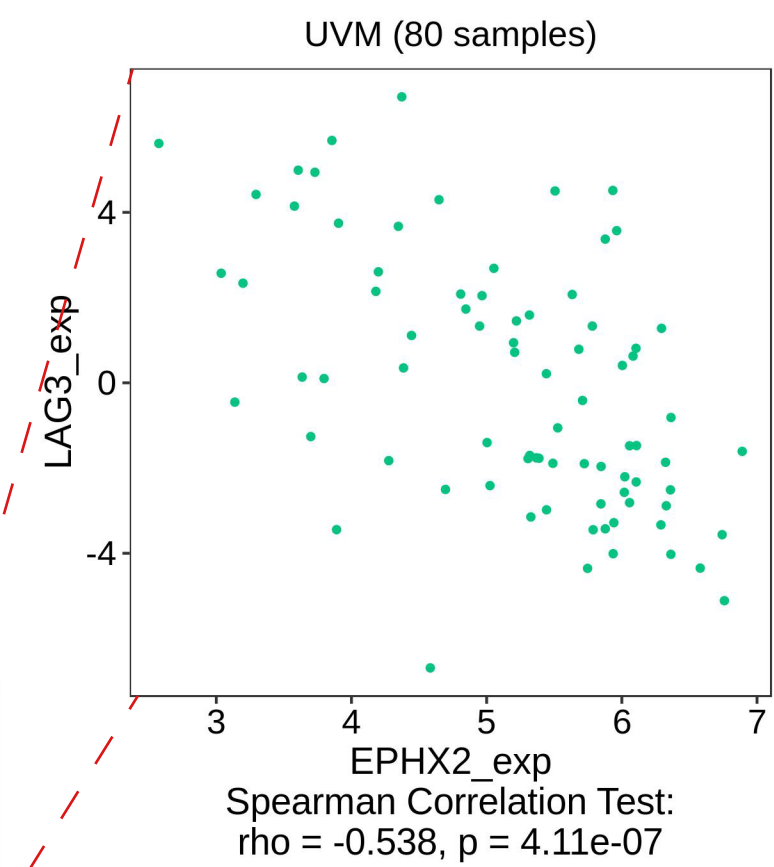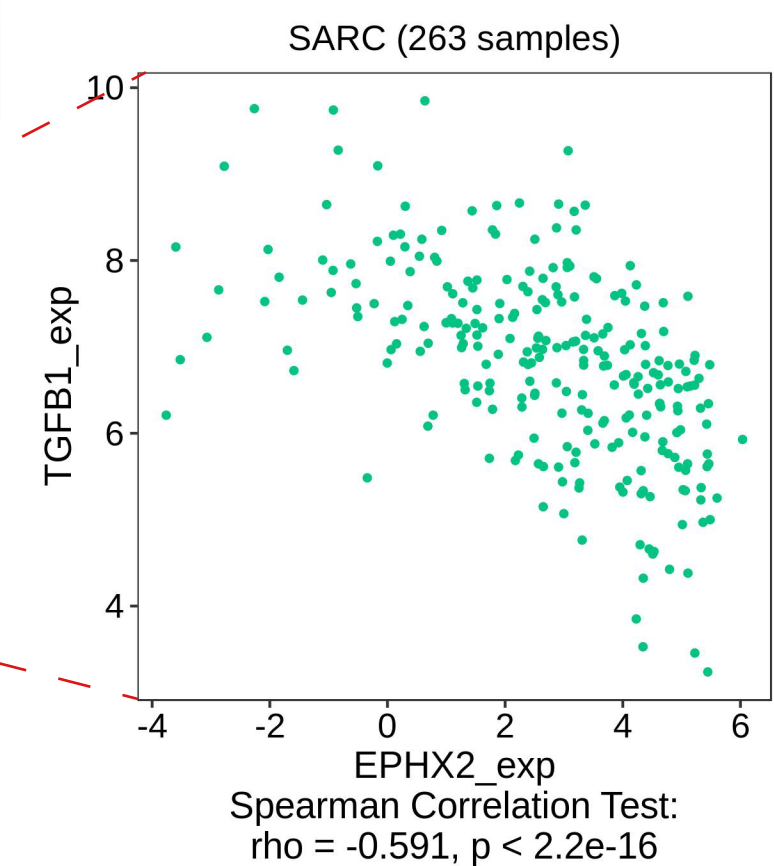

Supplement: Supplementary file 6 — Figure S6. The correlation between the EPHX2 expression and immune inhibitors. Red indicates positive correlation and blue indicates negative correlation. The first four strongest associations are shown by dot plots. [file CNR2-8-e70188-s002.pdf]

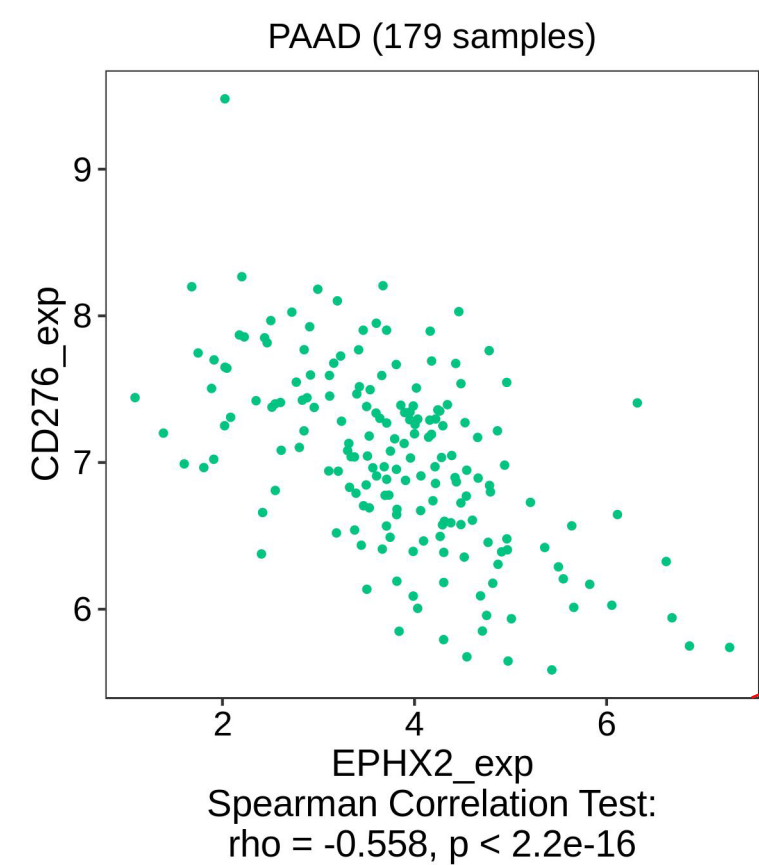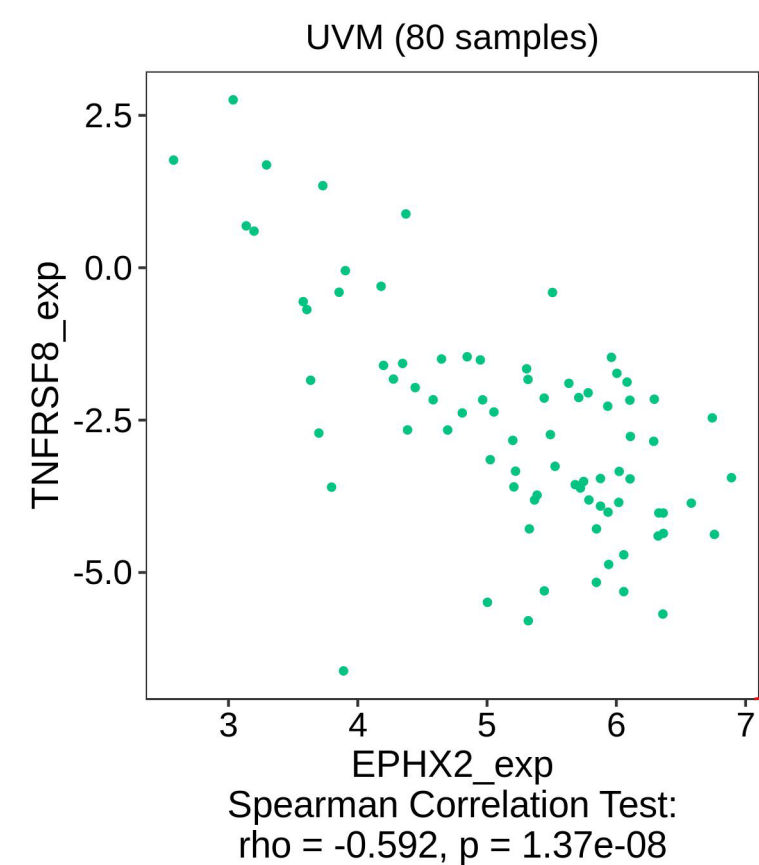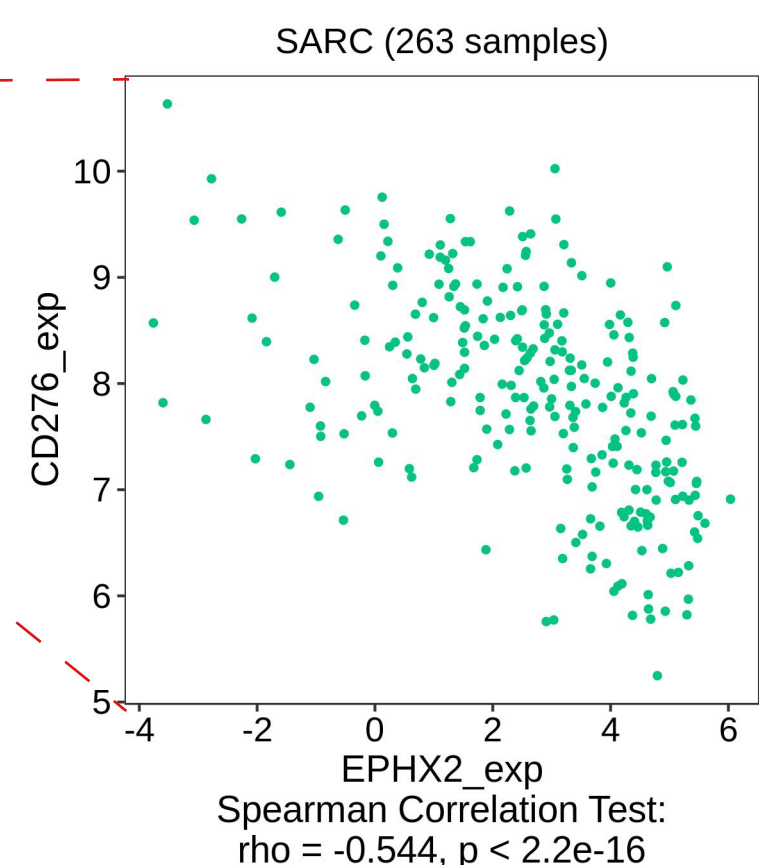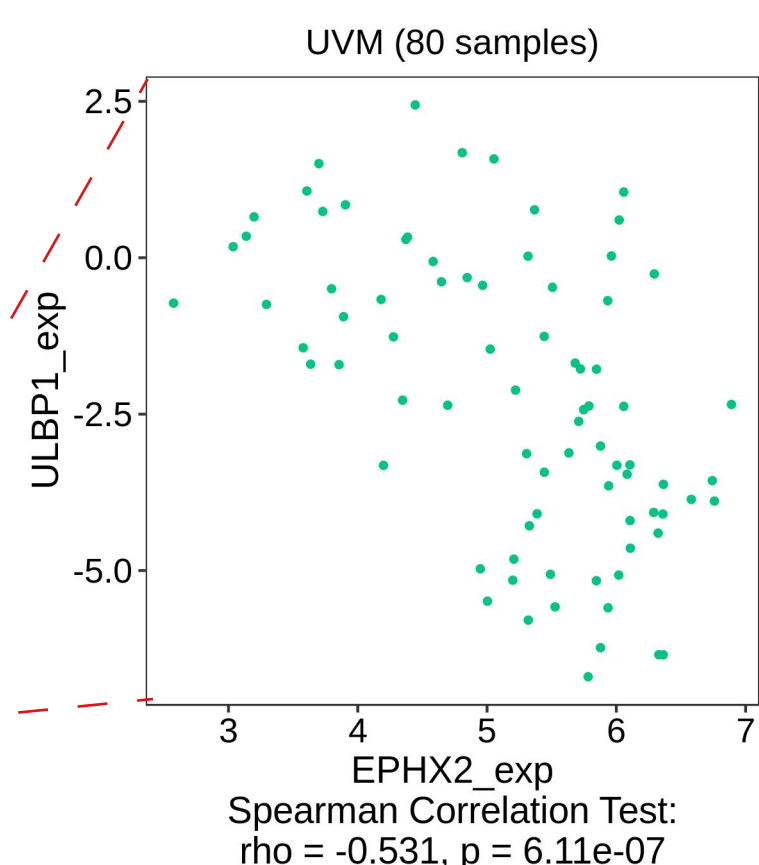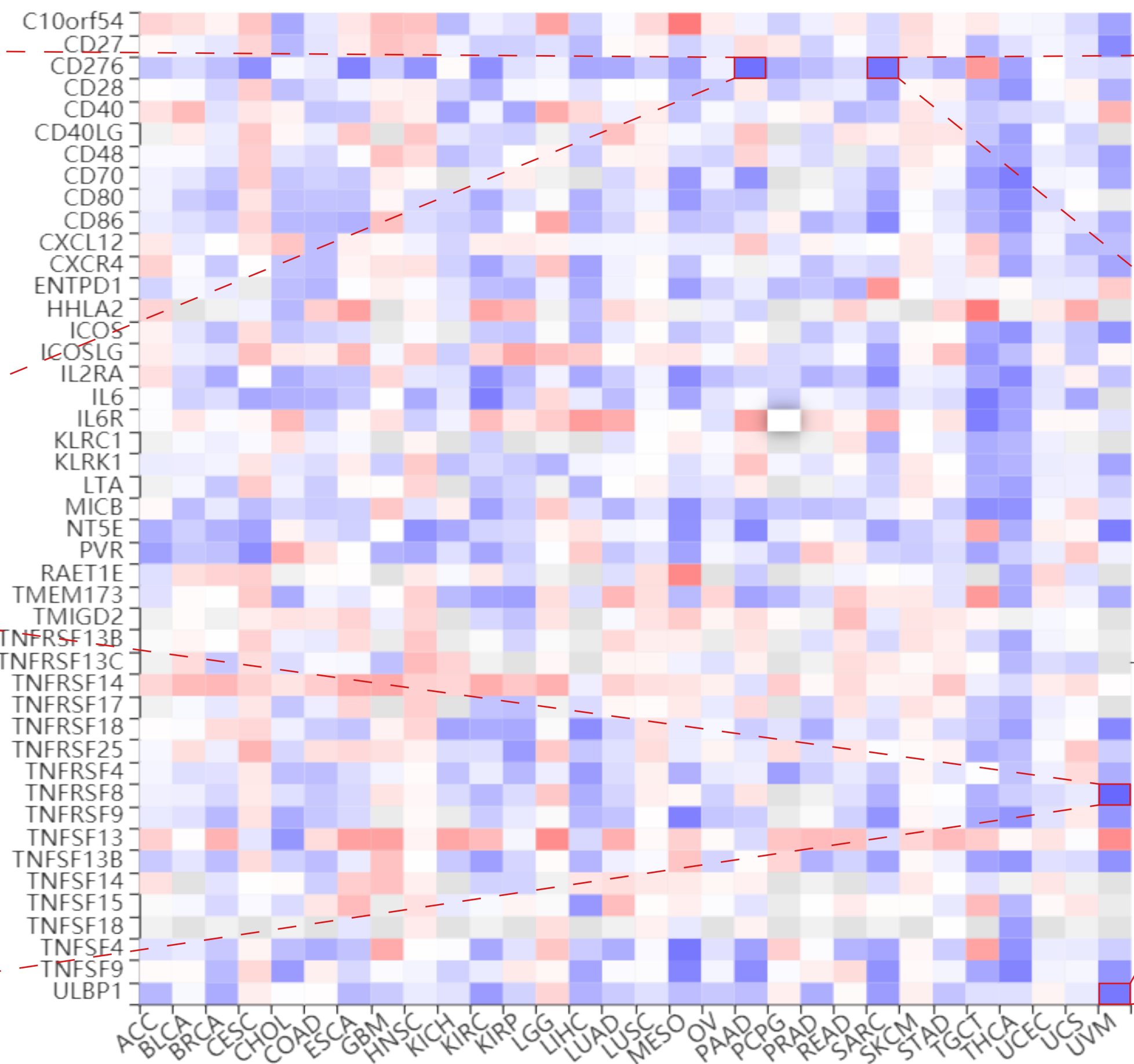

Supplement: Supplementary file 7 — Figure S7. The correlation between the EPHX2 expression and immune stimulators. Red indicates positive correlation and blue indicates negative correlation. The first four strongest associations are shown by dot plots. [file CNR2-8-e70188-s003.pdf]

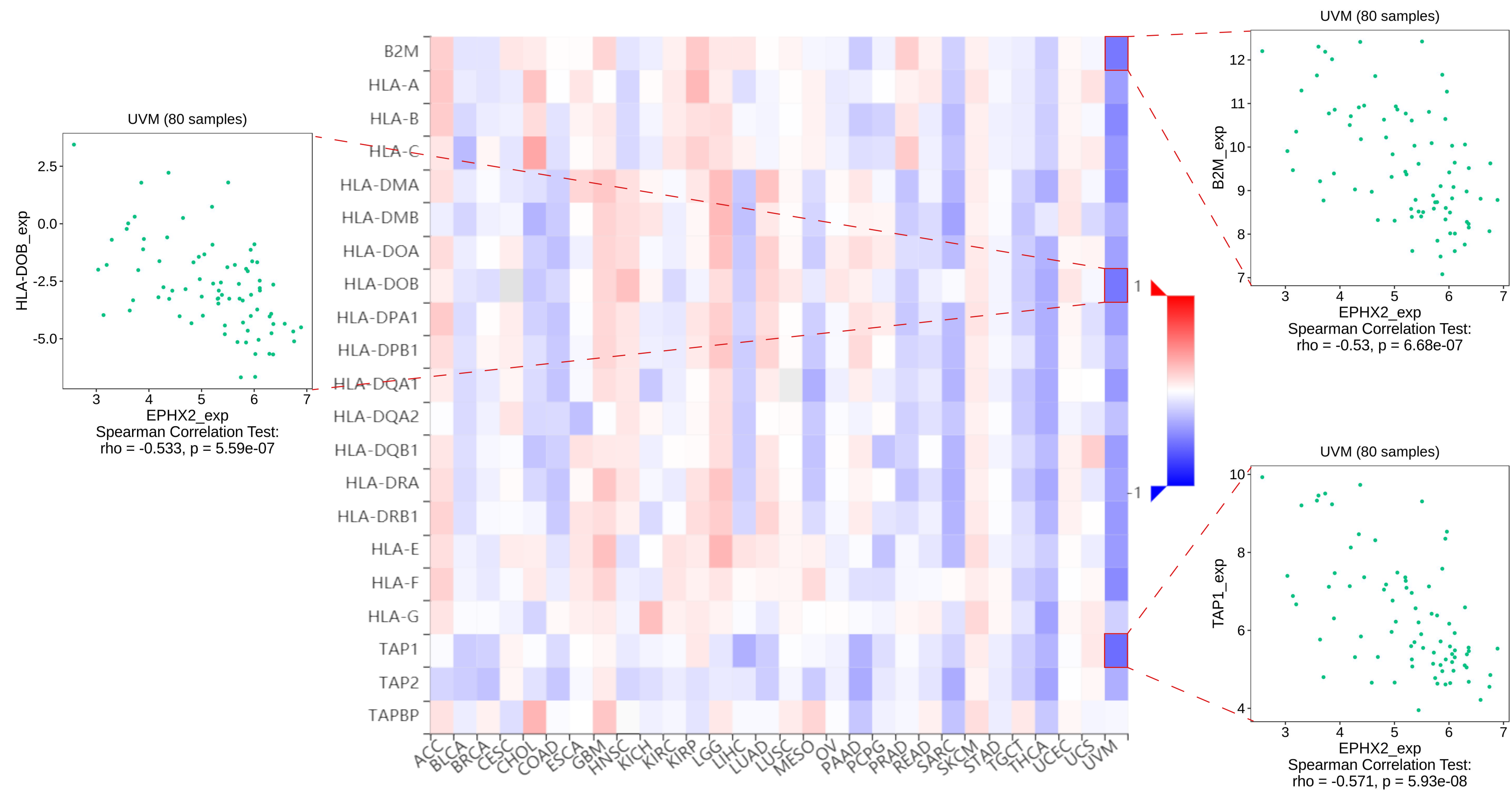

Supplement: Supplementary file 8 — Figure S8. The correlation between the EPHX2 expression and MHC molecules. Red indicates positive correlation and blue indicates negative correlation. The first three strongest associations are shown by dot plots. [file CNR2-8-e70188-s008.pdf]
