# Supplementary material for: A Comprehensive Analysis of Epoxide Hydrolase 2 (EPHX2) in Pan‐Cancer
Source: Cancer Rep (Hoboken). 2025 Mar 24;8(3):e70188. doi: 10.1002/cnr2.70188 (PMC11932960; doi:10.1002/cnr2.70188)
Supplement: Supplementary file 4 — Figure S4. Correlation between EPHX2 gene expression and TMB in TCGA database of KIRP, ESCA, BLCA, THCA, OV, LUAD, GBM, UCEC, PCPG, ACC, THYM, and DLBC. TMB, tumour mutational burden. [file CNR2-8-e70188-s001.pdf]

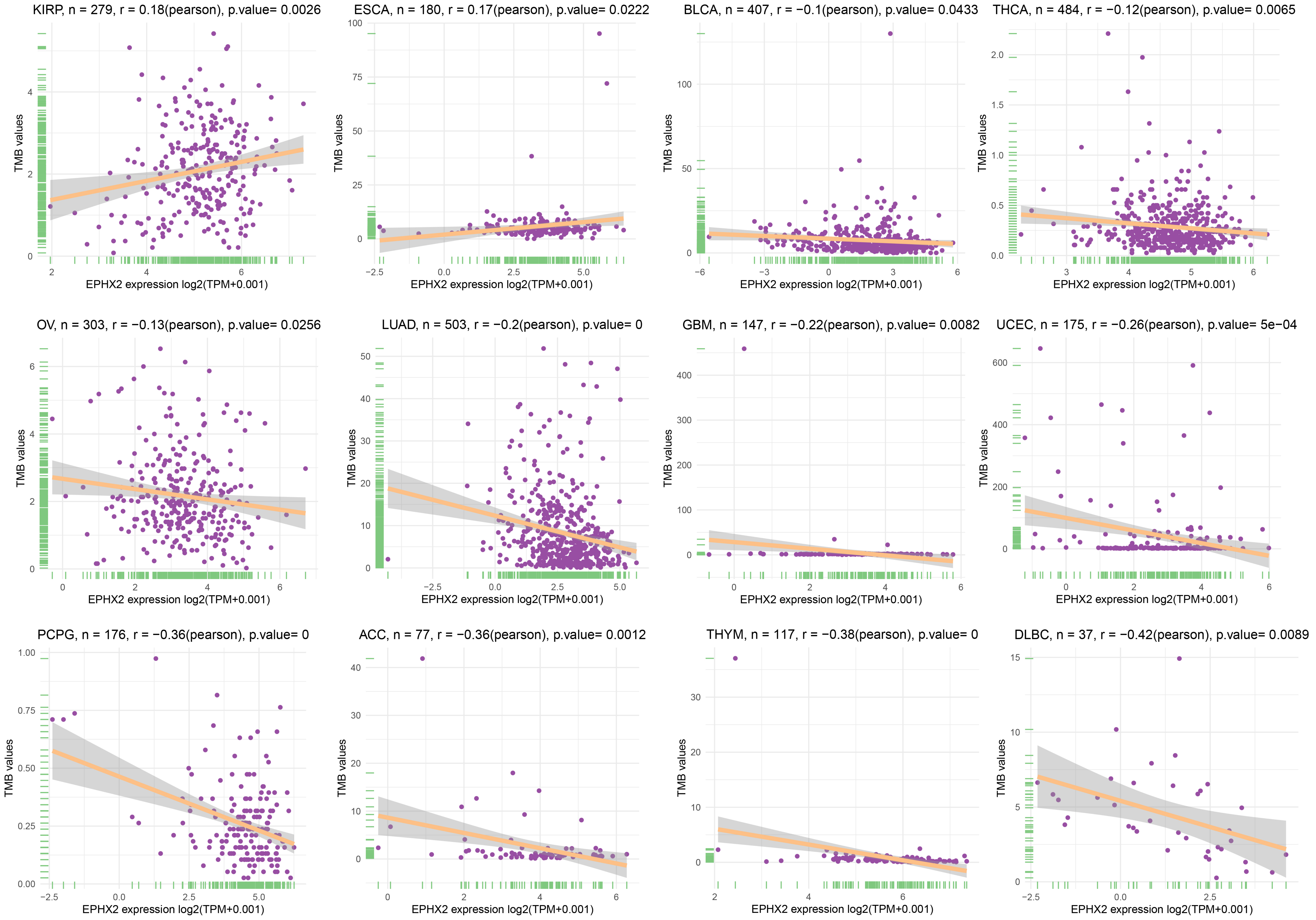
KIRP, n = 279, r = 0.18(pearson), p.value= 0.0026
TMB values
EPHX2 expression log2(TPM+0.001)
ESCA, n = 180, r = 0.17(pearson), p.value= 0.0222
TMB values
EPHX2 expression log2(TPM+0.001)
BLCA, n = 407, r = −0.1(pearson), p.value= 0.0433
TMB values
EPHX2 expression log2(TPM+0.001)
THCA, n = 484, r = −0.12(pearson), p.value= 0.0065
TMB values
EPHX2 expression log2(TPM+0.001)
OV, n = 303, r = −0.13(pearson), p.value= 0.0256
TMB values
EPHX2 expression log2(TPM+0.001)
LUAD, n = 503, r = −0.2(pearson), p.value= 0
TMB values
EPHX2 expression log2(TPM+0.001)
GBM, n = 147, r = −0.22(pearson), p.value= 0.0082
TMB values
EPHX2 expression log2(TPM+0.001)
UCEC, n = 175, r = −0.26(pearson), p.value= 5e−04
TMB values
EPHX2 expression log2(TPM+0.001)
PCPG, n = 176, r = −0.36(pearson), p.value= 0
TMB values
EPHX2 expression log2(TPM+0.001)
ACC, n = 77, r = −0.36(pearson), p.value= 0.0012
TMB values
EPHX2 expression log2(TPM+0.001)
THYM, n = 117, r = −0.38(pearson), p.value= 0
TMB values
EPHX2 expression log2(TPM+0.001)
DLBC, n = 37, r = −0.42(pearson), p.value= 0.0089
TMB values
EPHX2 expression log2(TPM+0.001)
